# Application of neurodegenerative disease treatment strategies in tinnitus: mechanisms, translation, and prospects

**DOI:** 10.3389/fnagi.2026.1835649

**Published:** 2026-05-29

**Authors:** Jingjing Liu, Peng Liu

**Affiliations:** Bethune International Peace Hospital, Shijiazhuang, China

**Keywords:** neurodegenerative diseases, neuroinflammation, synaptic plasticity, tinnitus, translational medicine, treatment strategies

## Abstract

Tinnitus, a prevalent auditory perception symptom, is closely associated with maladaptive neuroplasticity within the central auditory and non-auditory pathways. Its complex pathophysiology shares significant mechanistic parallels with neurodegenerative processes, including neuroinflammation, synaptic dysfunction, excitotoxicity, and aberrant neural network reorganization. This review explores the therapeutic potential of repurposing strategies originally developed for neurodegenerative diseases for tinnitus intervention. We systematically examine key approaches, such as targeting neuroinflammatory cascades, modulating neurotrophic factors, and mitigating glutamate-mediated excitotoxicity. The discussion synthesizes evidence from preclinical studies suggesting their mechanisms of action within tinnitus models and evaluates the current landscape of translational and clinical research. By bridging insights from neurodegeneration and auditory neuroscience, this article aims to provide a cohesive theoretical framework and identify future directions for developing novel, mechanism-based therapeutic interventions for tinnitus.

## 1 Introduction

Tinnitus, the perception of sound in the absence of an external acoustic source, is a complex and often distressing symptom affecting a significant portion of the global population (Jarach et al., 2022). Transcriptomic studies of central auditory structures including the auditory cortex (AC), inferior colliculus (IC), and cochlear nucleus (CN) have identified neuronal hyperactivity, oxidative stress, and neuroinflammation in tinnitus pathophysiology (Song et al., 2026). Understanding tinnitus as a progressive disorder that is initiated peripherally, transmitted and amplified through the brainstem and midbrain, and finally consolidated in the cortex and associated networks is crucial for precisely locating therapeutic targets. Tinnitus often begins with damage to cochlear hair cells or the auditory nerve (such as noise and ototoxic drugs) (Chu et al., 2020; Song et al., 2026). This peripheral deafferentation, which reduces normal signal input to the central auditory system, is the initial driver for a series of subsequent central compensatory changes and remodeling (Auerbach et al., 2014). As the first relay stations for auditory information entering the central nervous system, brainstem structures like the CN are among the first to undergo neural plasticity. This stage is mainly characterized by a compensatory “central gain” increase, where neurons become hyper-responsive to diminished peripheral inputs to compensate for the signal loss (Auerbach et al., 2014; Eggermont, 2024). The IC, as a major auditory integration center in the midbrain, shows significant neuroinflammatory activation and metabolic changes in tinnitus models (Olthof et al., 2022; Ahmed and Ahmed, 2022). It receives and integrates the upregulated signals from the brainstem. Concurrently, a disruption in excitatory/inhibitory (E/I) balance may occur here, potentially due to weakened function of inhibitory neurotransmitters (Olthof et al., 2022). At this stage, aberrant neural activity is significantly amplified and relayed, setting the stage for cortical perception. The AC (particularly the primary A1) is the key region where the tinnitus percept is ultimately formed and consolidated (Ahmed and Ahmed, 2022; De Ridder et al., 2014). Chronic tinnitus is closely associated with increased spontaneous neuronal firing rates, broadened tuning curves, and “map reorganization” in the cortical area representing the damaged frequency in AC (De Ridder et al., 2014; Eggermont, 2006). This aberrant synaptic plasticity and network reorganization creates a stubborn “tinnitus trace” or memory-like representation in the cortex, allowing the phantom perception to persist even after the initial peripheral damage may have healed (Eggermont, 2006; Husain and Khan, 2023). The chronic and distressing nature of tinnitus involves the deep engagement of non-auditory brain regions. The limbic system (e.g., amygdala, hippocampus) assigns negative emotional valence to tinnitus; the prefrontal cortex, involved in attention and cognitive control, becomes dysregulated, preventing patients from ignoring the tinnitus (De Ridder et al., 2014; Lan et al., 2022; Singh et al., 2023). Consequently, chronic tinnitus ultimately evolves into a distributed neural network disorder involving auditory, limbic, and attentional networks (Husain and Khan, 2023; Sedley, 2019).

The chronicity of tinnitus is increasingly recognized not merely as a static condition but as a process involving progressive alterations within the central nervous system. A growing body of epidemiological and mechanistic evidence suggests that these persistent changes share remarkable similarities with the pathophysiological hallmarks of classical neurodegenerative disorders like Alzheimer’s disease (AD) and Parkinson’s disease (PD). For instance, a large-scale retrospective cohort study utilizing the Taiwanese National Health Insurance Research Database demonstrated that patients with tinnitus had a significantly higher risk of subsequently developing AD (adjusted hazard ratio 1.54) and PD (adjusted hazard ratio 1.56) over a 10-year follow-up period, positioning tinnitus as a potential “soft” sign or risk factor for these neurodegenerative conditions (Chu et al., 2020). This association underscores a possible shared etiological landscape beyond mere comorbidity.

The mechanistic parallels between chronic tinnitus and neurodegeneration are multifaceted. Key pathological processes common to both include chronic neuroinflammation, oxidative stress, mitochondrial dysfunction, and, in some studies, suggestive evidence of aberrant protein aggregation, and disruptions in the delicate balance between excitatory and inhibitory neurotransmission (Shulman et al., 2021; Lai et al., 2024; Lazzeri et al., 2023; Marschall et al., 2020). Transcriptomic analyses in animal models of noise-induced tinnitus have revealed significant alterations in gene expression profiles within the AC, with pathway enrichment analyses indicating increased activity in pathways related to neurodegenerative disorders such as Huntington’s disease (HD) and Amyotrophic lateral sclerosis(ALS) (Liu et al., 2023). Furthermore, oxidative stress emerges as a central player. Metabolomic studies in tinnitus models have revealed significant enrichment of oxidative stress-related pathways, including glutathione metabolism, in the AC (Yang et al., 2025). The critical role of the redox-sensitive transcription factor Nrf2 is highlighted by findings that Nrf2-deficient mice exhibit increased susceptibility to prolonged, noise-induced tinnitus-like behavior, accompanied by heightened microglial activation and neuroinflammation (Yang et al., 2025). This oxidative milieu is not confined to animal models; in Meniere’s disease, considered a model of cochlear neurodegeneration, patients exhibit systemic oxidative stress, which can be modulated by interventions targeting cellular stress response pathways (Scuto et al., 2020).

Beyond molecular and cellular mechanisms, structural and functional system-level impairments further cement this connection. Notably, the glymphatic system, a recently characterized brain-wide waste clearance pathway pivotal for the removal of metabolic by-products like amyloid-*β* and whose dysfunction is heavily implicated in neurodegenerative diseases, shows significant impairment in chronic tinnitus patients (Du et al., 2025). Using diffusion tensor imaging along the perivascular space (DTI-ALPS), studies have found a significantly lower glymphatic index in tinnitus patients compared to healthy controls, and this decrease correlated with poorer performance on specific cognitive tasks (Du et al., 2024). This provides a direct link between tinnitus, impaired brain clearance mechanisms, and cognitive deficits, hallmarks of neurodegenerative progression (Zhang and Liao, 2025; Rosická et al., 2024; Yang et al., 2024; Gasparre et al., 2023). Additionally, evidence of axonal degeneration is found in peripheral neural structures. Evaluations in unilateral tinnitus patients have demonstrated a negative correlation between cochlear nerve fiber thickness and both hearing loss and tinnitus severity, with similar relationships observed in retinal nerve fiber layer thickness, suggesting a possible underlying neurodegenerative component in the disease etiology (Avcu et al., 2021). The involvement of E/I imbalance, a core feature in several neurological disorders, is also well-documented in tinnitus (Isler et al., 2022; Schaette and McAlpine, 2011; Yang et al., 2007). Stress, a known exacerbating factor, can induce tinnitus-like behavior in animal models, linked to decreased expression of GABA A receptor α1 in the hippocampus, indicating a shift towards neuronal hyperexcitability. Pharmacological modulation of this imbalance, such as with Acamprosate, which affects neurotransmission in the nucleus accumbens, can partially reverse salicylate-induced tinnitus and associated molecular changes in N-methyl-D-aspartate (NMDA) and GABA receptor subunits (Farrahizadeh et al., 2025).

**Figure 1:**
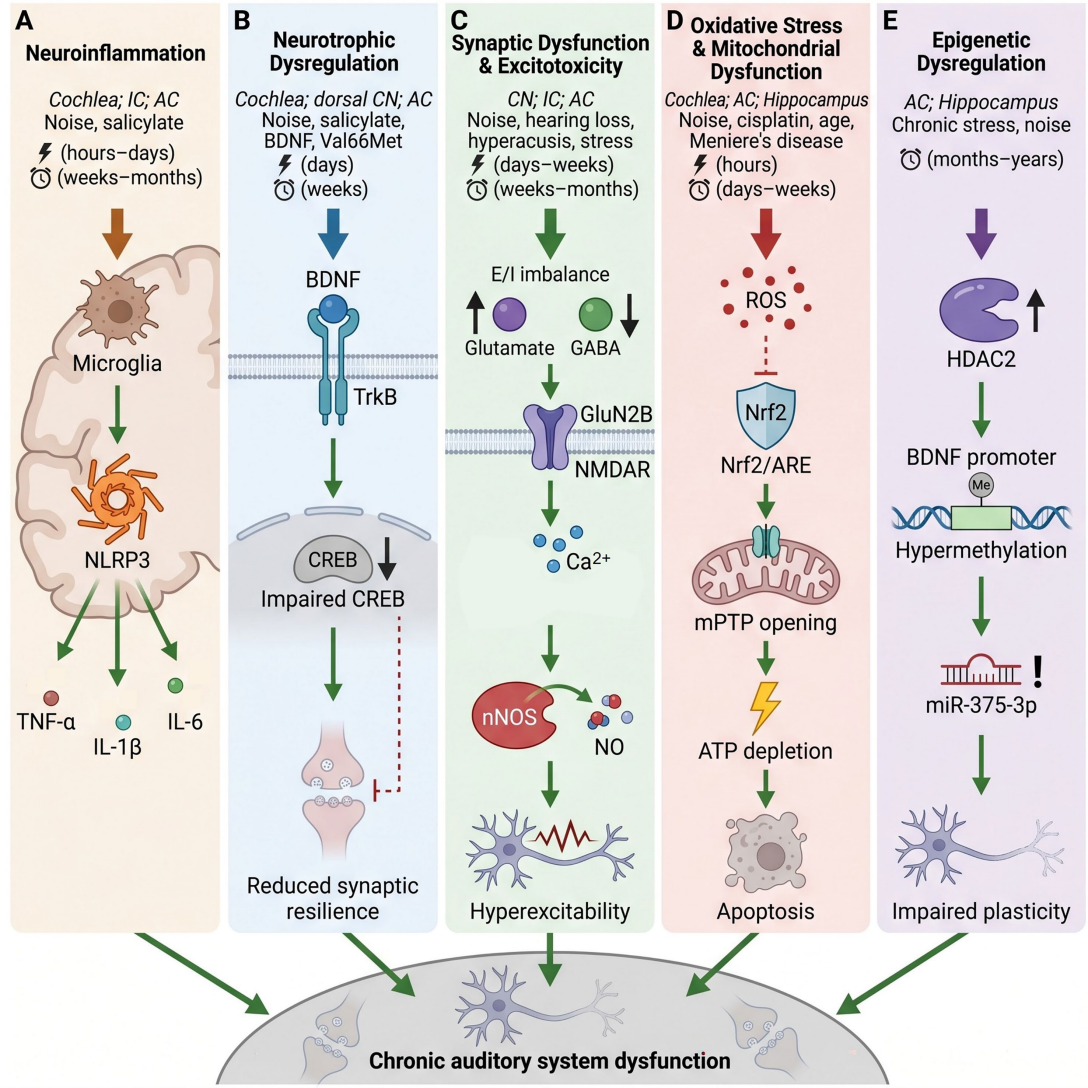
Shared pathological mechanisms in tinnitus and neurodegenerative diseases. This figure summarizes five core pathological pathways **(**column **A–E)** linking tinnitus to neurodegenerative disorders. The upper part of each column lists the primary brain regions involved, the main causative insults, and the typical time scale. The lower part shows the pathological cascade leading to neuronal dysfunction. All mechanisms are discussed in the corresponding sections of the manuscript (see Section 2 for mechanisms). Neuroinflammation **(A)**: Activated microglia trigger the NLRP3 inflammasome, releasing TNF-*α*, IL-1β, and IL-6. Predominant in AC, IC, and cochlea. Caused by noise or salicylate. Neurotrophic dysregulation **(B)**: Reduced BDNF/TrkB signaling impairs CREB and synaptic resilience. Affects cochlea, dorsal CN, and AC. Causes include noise, salicylate, and BDNF Val66Met polymorphism. Synaptic dysfunction and excitotoxicity **(C)**: Excitatory/inhibitory imbalance (↑glutamate, ↓GABA) leads to NMDAR overactivation (GluN2B), Ca^2+^ overload, nNOS/NO signaling, and hyperexcitability. Involves CN, IC, and AC. Triggered by noise, hearing loss, hyperacusis, and stress. Oxidative stress and mitochondrial dysfunction **(D)**: ROS overproduction suppresses the Nrf2/ARE pathway, opens mPTP, depletes ATP, and causes apoptosis. Damages cochlea, AC, and hippocampus. Causes include noise, cisplatin, ageing, and Meniere’s disease. Epigenetic dysregulation **(E)**: HDAC2 overactivity, BDNF promoter hypermethylation, and dysregulated miRNAs (e.g., miR-375-3p) produce durable changes. Mainly in AC and hippocampus. Induced by chronic stress and noise.

**Figure 2:**
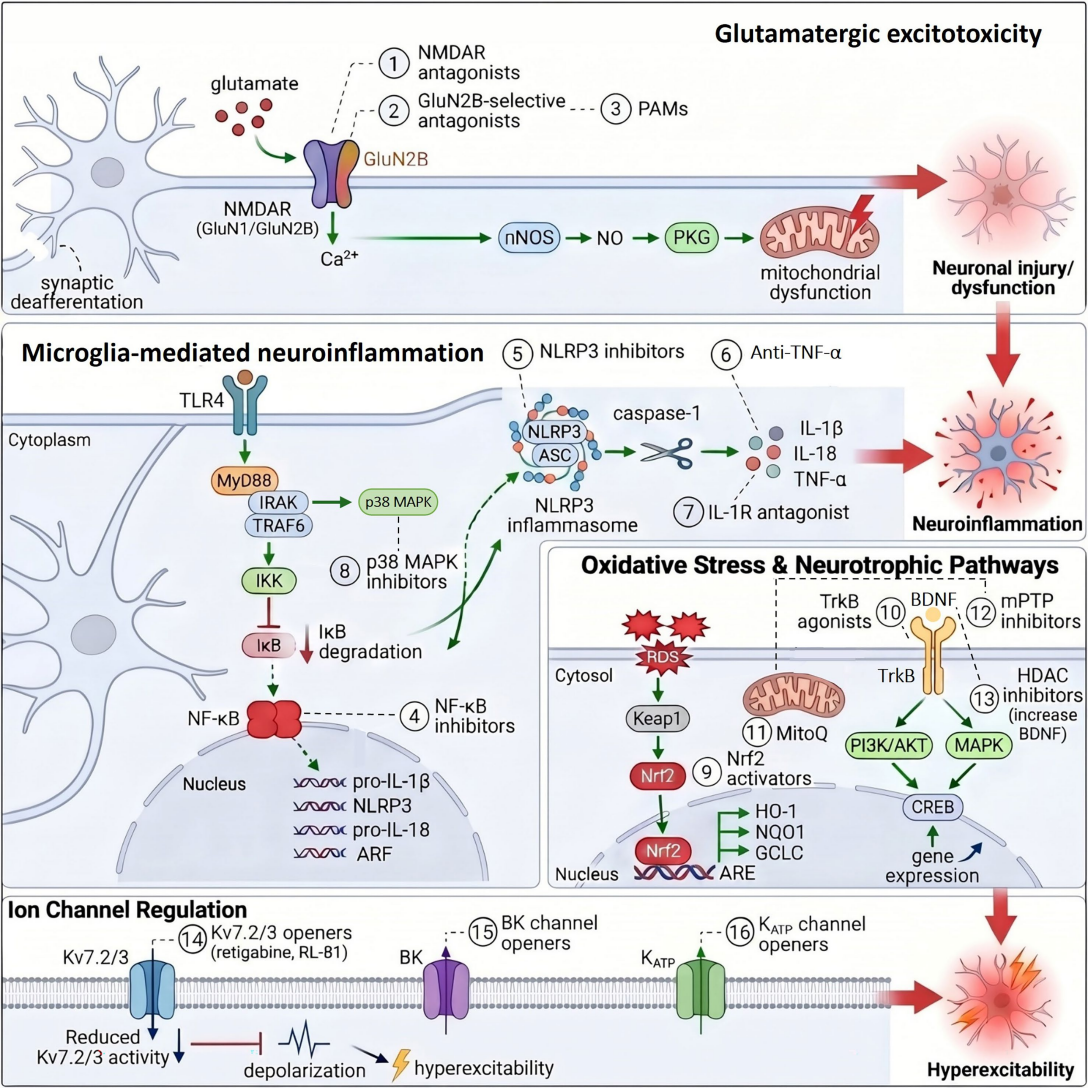
Detailed molecular signaling pathways and druggable targets in tinnitus. This figure maps the cascades of glutamatergic excitotoxicity, microglia-mediated neuroinflammation, oxidative stress and neurotrophic signaling, and ion channel regulation. Numbered intervention nodes (①–⑯) correspond to the therapeutic agents or strategies. Glutamatergic excitotoxicity panel: Peripheral deafferentation → glutamate release → NMDAR (GluN1/GluN2B) → Ca^2+^ influx → nNOS → NO → PKG → mitochondrial dysfunction and neuronal injury. Nodes: ① NMDAR antagonists; ② GluN2B-selective antagonists; ③ positive allosteric modulators (PAMs). Microglia-mediated neuroinflammation panel: TLR4 activation → MyD88/IRAK/TRAF6 and p38 MAPK → IKK → IκB degradation → NF-κB nuclear translocation → transcription of pro-IL-1β, NLRP3, pro-IL-18 → NLRP3 inflammasome assembly (NLRP3/ASC/pro-caspase-1) → caspase-1 → mature IL-1β, IL-18, TNF-*α*. Nodes: ④ NF-κB inhibitors; ⑤ NLRP3 inhibitors; ⑥ anti-TNF-*α* antibodies; ⑦ IL-1 receptor antagonist; ⑧ p38 MAPK inhibitors. Oxidative stress and neurotrophic pathways panel: Oxidative branch: ROS → Keap1 oxidation → Nrf2 release → nuclear translocation → ARE binding → expression of HO-1, NQO1, GCLC. Neurotrophic branch: BDNF → TrkB → PI3K/AKT and MAPK signaling → CREB → gene expression. Nodes: ⑨ Nrf2 activators; ⑩ TrkB agonists; ⑪ MitoQ (mitochondrial antioxidant); ⑫ mPTP inhibitors; ⑬ HDAC inhibitors (increase BDNF expression). Ion channel regulation panel: Reduced Kv7.2/3 channel activity → membrane depolarization → neuronal hyperexcitability. Nodes: ⑭ Kv7.2/3 openers (retigabine, RL-81); ⑮ BK channel openers (BMS-191011); ⑯ K_ATP_ channel openers (diazoxide). Symbols: Arrows (→) indicate activation; T-bars (—|) indicate inhibition. Numbered circles are connected by dashed lines to their molecular targets. All pathways and nodes are supported by evidence cited in the main text.

The convergence of these pathways suggests that chronic tinnitus may represent a localized or system-wide prodromal or concomitant neurodegenerative process within the central auditory and associated networks. This perspective is bolstered by the observation that interventions developed for neurodegenerative diseases sometimes intersect with tinnitus symptomatology, as seen in a meta-analysis of gamma-frequency stimulation for AD, which, while aiming to improve cognition, was associated with an increased risk of inducing tinnitus as a side effect (Ang et al., 2025). Conversely, agents with neuroprotective properties used in traditional medicine for conditions like forgetfulness and tinnitus, such as Hawthorn Leaf Flavonoids, show efficacy in AD models by reducing oxidative stress and neuroinflammation via the Nrf-2 pathway, indicating common protective mechanisms (Xu et al., 2023). Therefore, reconceptualizing chronic tinnitus through the lens of neurodegenerative biology provides a compelling framework for understanding its persistence and progression. It argues for a strategic shift in therapeutic development, advocating for the repurposing and adaptation of neuroprotective, anti-inflammatory, and redox-modulating strategies proven or proposed in AD, PD, and other neurodegenerative diseases. This approach holds significant promise for moving beyond symptomatic sound-based management towards interventions that target the underlying progressive neuropathological cascade, potentially altering the disease course and improving long-term outcomes for patients with debilitating chronic tinnitus. To compile evidence for this narrative review, a multi-tiered literature search strategy was employed. First, a systematic search was conducted in PubMed, Web of Science, and Embase from inception to March 2026 using combinations of “tinnitus” AND (“neurodegenerative diseases” OR “Alzheimer disease” OR “Parkinson disease”) AND (“treatment” OR “therapy” OR “intervention”), supplemented with mechanism-specific terms paired with “tinnitus” (e.g., “neuroinflammation,” “oxidative stress,” “BDNF,” “NMDA receptor,” “epigenetic”). Second, given the central aim of this review which is to bridge insights from neurodegeneration and tinnitus. We identified shared pathological pathways and therapeutic targets for which direct tinnitus evidence remains limited (e.g., HDAC inhibition, GDNF, NLRP3 inflammasome, mitochondrial transplantation). For these, targeted searches were performed in PubMed using the pathway or target term combined with “neurodegeneration” or specific disease names (e.g., “HDAC inhibitor AND Huntington’s disease”) without the “tinnitus” keyword, and the translational relevance was evaluated by the authors. Third, to ensure comprehensive coverage of therapeutic mechanisms and safety considerations, we conducted complementary searches in the broader neuroscience, pharmacology, and oncology literature (e.g., BDNF in cancer, optogenetic tools). Throughout the process, reference lists of key papers and major reviews were hand-searched. We included peer-reviewed original articles, systematic reviews, and clinical trials in English. Studies on hearing loss without tinnitus, case reports, and editorials were excluded. Two reviewers screened titles, abstracts, and full texts. No formal quality scoring was performed, given the narrative nature of the review. The shared pathological mechanisms discussed below are graphically summarized in Figure 1, and the corresponding molecular targets and druggable nodes are illustrated in Figure 2.

## 2 Shared pathological mechanisms underlying tinnitus and neurodegenerative diseases

Current evidence supports a multi-stage, multi-pathway hypothesis for tinnitus generation. Tinnitus is generated centrally in the auditory pathway, with neural recordings in animal models revealing increased spontaneous firing rates in the auditory brainstem, midbrain, and forebrain (Hockley and Shore, 2019). The mechanisms proposed to explain this increase include spike-timing-dependent plasticity, homeostatic plasticity, central gain, reduced inhibition, thalamocortical dysrhythmia, and increased inflammation (Hockley and Shore, 2019). Tinnitus typically begins with peripheral auditory injury (noise, ototoxins, or age-related cochlear damage). This injury reduces afferent input, triggering a cascade of compensatory central plasticity (Roberts, 2018). Auditory neurons compensate for deafferentation by increasing their input/output functions (gain) at multiple levels of the auditory system. Forms of homeostatic plasticity increase the spontaneous and driven activity of neurons in central auditory structures in animals expressing behavioral evidence of tinnitus (Roberts, 2018).

If peripheral injury persists or the compensatory response becomes maladaptive, secondary pathological processes emerge and consolidate the tinnitus percept. These include: chronic neuroinflammation – noise-induced hearing loss is associated with elevated proinflammatory cytokines and microglial activation in the primary AC; pharmacological depletion of microglia prevents noise-induced tinnitus (Wang et al., 2019); dysregulated neurotrophic support – salicylate-induced tinnitus is associated with altered BDNF/TrkB/CREB signaling in the AC (Yi et al., 2018); oxidative stress – Nrf2-deficient mice display increased susceptibility to noise-induced tinnitus-like behavior, accompanied by heightened microglial activation and neuroinflammation (Yang et al., 2025); and epigenetic modifications – DNA methylation alterations have been identified in tinnitus patients, suggesting epigenetic underpinnings (Bhatt et al., 2024).

These pathways do not operate in isolation. Oxidative stress triggers neuroinflammation via NF-κB activation, and neuroinflammation further impairs BDNF/TrkB signaling (Yang et al., 2025). The end result is a self-sustaining, network-level disorder involving not only the AC but also limbic regions and prefrontal cortex, which assign emotional valence and attentional salience to the phantom sound (De Ridder et al., 2014). Critically, the relative contribution of each pathway depends on underlying cause (noise-induced vs. drug-induced), brain region (cochlear vs. brainstem vs. cortical vs. limbic), and time scale (acute/initiation vs. chronic/maintenance).

### 2.1 Sustained neuroinflammation and glial cell activation

In tinnitus, persistent activation of microglia and astrocytes is observed, accompanied by elevated levels of pro-inflammatory cytokines tumor necrosis factor-alpha (TNF-*α*) and interleukin-1β (IL-1β) (Mennink et al., 2022). This chronic neuroinflammatory state, characterized by glial reactivity, shares significant similarities with the neuroinflammatory hallmarks observed in neurodegenerative conditions such as AD, where sustained glial activation contributes to progressive neural dysfunction. The activation process is evident in various tinnitus models. For instance, in salicylate-induced tinnitus rats, significant upregulation of the microglial marker Iba1 occurs in the primary AC and medial geniculate body, and significant upregulation of the astrocytic marker GFAP occurs in the primary AC, alongside increased morphological complexity indicative of an activated state (Xia et al., 2020). Importantly, the causal role of microglia-driven neuroinflammation in tinnitus pathophysiology is most strongly established in noise-induced tinnitus models (Wang et al., 2019; Luo et al., 2024). Wang et al. (2019) demonstrated that noise-induced hearing loss in mice is associated with elevated proinflammatory cytokines and microglial activation in the primary AC, and that genetic knockout of TNF-*α* or pharmacological depletion of microglia prevented the development of tinnitus-related behavioral phenotypes. Critically, direct infusion of TNF-*α* into the AI of normal-hearing mice induced tinnitus-like behavioral signs, providing causal evidence that microglial activation and TNF-*α* signaling are sufficient to drive tinnitus pathophysiology (Wang et al., 2019). The involvement of the NLRP3 inflammasome pathway has also been implicated, with pharmacological inhibition of TLR4/NF-κB/NLRP3 signaling attenuating neuroinflammation and improving tinnitus behavior in noise-exposed mice (Luo et al., 2024). In contrast, while salicylate-induced tinnitus models show significant astrocyte and microglial activation in the AC and medial geniculate body, including upregulation of GFAP and Iba1 alongside increased IL-1β expression (Xia et al., 2020), the causal dependence on microglial activation in this model remains less certain—some evidence suggests that salicylate-induced tinnitus may persist even when microglial activation is pharmacologically suppressed (Glia, 2015). These findings suggest that neuroinflammation is a common pathway across multiple tinnitus etiologies, but the degree to which microglia serve as an indispensable driver may vary by insult type. Within the auditory pathway, the most robust evidence for glial-driven neuroinflammation localizes to the AC, where microglial activation directly modulates excitatory-inhibitory balance and synaptic transmission. Evidence of neuroinflammation in the cochlea is also present, including noise-induced activation of NLRP3 inflammasome-mediated inflammatory cascades, but the precise contribution of glial-like cells (e.g., macrophages) in the peripheral auditory system to tinnitus generation remains less well characterized (see Figure 1A) for a schematic of the neuroinflammatory cascade (Mennink et al., 2022).

An important unresolved question is whether neuroinflammation primarily drives the initiation of tinnitus or contributes to its chronic persistence. Based on available evidence from rodent models, we hypothesize that the role of neuroinflammation follows a biphasic, etiology-dependent temporal profile.

In noise-induced tinnitus models, neuroinflammation serves as an early initiating driver. TNF-*α* mRNA levels rise rapidly within 12 h of noise exposure, and microglial activation, evidenced by deramification and increased soma-to-whole cell size ratio, becomes significant by day 5 post-exposure (Wang et al., 2019; Luo et al., 2024). Critically, TNF-*α* infusion into the primary AC of normal-hearing mice directly induces tinnitus-like behaviors, establishing causality for the initial phase (Wang et al., 2019). Moreover, pharmacological blockade of TNF-*α* even at 7–10 days post-noise exposure can ameliorate tinnitus behavior, suggesting that sustained neuroinflammation contributes to chronic maintenance of the tinnitus state (Wang et al., 2019). This biphasic pattern—early acute inflammatory signaling followed by persistent low-grade neuroinflammation—parallels mechanisms observed in chronic pain and other neuroinflammatory disorders.

In contrast, salicylate-induced tinnitus exhibits a distinct temporal profile. Salicylate administration induces transient tinnitus that emerges within 2 h, persists throughout the treatment period, and resolves within 24 h of drug cessation (Ralli et al., 2010). Salicylate-induced increases in TNF-*α* and NR2A expression in the cochlear nucleus return to baseline 14 days after treatment withdrawal (Hu et al., 2014). Furthermore, salicylate-induced tinnitus does not appear to require microglial activation, as minocycline-mediated microglial suppression fails to block tinnitus behavior in this model (Perin et al., 2015). These findings suggest that in salicylate-induced tinnitus, inflammation plays a role that is tightly coupled to drug exposure rather than driving autonomous chronic pathology.

Within the auditory pathway, the most robust evidence for time-dependent neuroinflammation localizes to the primary AC, where early microglial activation and sustained cytokine elevation have been documented (Wang et al., 2019; Luo et al., 2024). In contrast, evidence for chronic neuroinflammation in subcortical structures such as the CN remains more limited, although reversible inflammatory gene expression changes have been reported in the IC and MGB in salicylate models (Xia et al., 2020; Hu et al., 2014; Hwang et al., 2011).

Together, these observations support the hypothesis that in noise-induced tinnitus—the model most relevant to the majority of human cases—neuroinflammation acts as both an acute trigger (within hours to days post-insult) and a chronic perpetuator (over weeks to months), whereas in pharmacologically induced (salicylate) tinnitus, inflammation is a reversible correlate of the acute state rather than a driver of chronicity. This distinction has important implications for therapeutic timing: anti-inflammatory interventions may be most effective if initiated early post-insult to prevent the establishment of chronic tinnitus, but may also confer benefit in established tinnitus by reducing sustained inflammatory signaling that perpetuates maladaptive plasticity.

### 2.2 Dysregulation of neurotrophic support

Brain-derived neurotrophic factor (BDNF) and its high-affinity receptor, tropomyosin receptor kinase B (TrkB), constitute a signaling axis fundamental to neuronal survival, differentiation, and synaptic plasticity within the central nervous system (Numakawa and Kajihara, 2025). Within the auditory system, BDNF is expressed in the inner and outer hair cells and spiral ganglion neurons of the cochlea, whereas TrkB is expressed in the spiral ganglion, establishing this pathway as critical for auditory neuronal development and maintenance (Pisani et al., 2023). Auditory activity bidirectionally regulates BDNF and TrkB expression in the AC, as bilateral cochlear ablation induces dynamic changes that initially decrease at 2 weeks but increase at 4 weeks post-ablation, followed by reduction at 6 and 8 weeks, demonstrating that sensory input directly modulates BDNF/TrkB signaling in central auditory structures (Wang et al., 2017).

Direct evidence linking BDNF/TrkB signaling to tinnitus has emerged from both salicylate-induced and noise-induced models. In a salicylate-induced tinnitus rat model, chronic systemic salicylate administration significantly upregulated BDNF expression and CREB phosphorylation in the AC, accompanied by ultrastructural synaptic changes (Yi et al., 2018). These alterations returned to normal after a 14-day recovery period, indicating a reversible relationship between BDNF signaling and salicylate-induced tinnitus (Yi et al., 2018). Critically, therapeutic modulation of this pathway has demonstrated behavioral efficacy: electrical stimulation at auricular points combined with sound masking improved tinnitus-related behavioral measures (GPIAS ratios) in salicylate-induced tinnitus rats, and this effect was associated with reversal of BDNF/TrkB/CREB signaling pathway overactivation in the AC (Yang et al., 2019). Moreover, local application of the GABAA receptor modulator midazolam to the cochlea reversed salicylate-induced increases in cochlear BDNF expression while concurrently reducing tinnitus perception in the animal model, accompanied by restoration of cortical Arg3.1 expression, revealing a functional BDNF-mediated cochlear–cortical axis in tinnitus pathophysiology (Panford-Walsh et al., 2008).

In noise-induced tinnitus models, complementary evidence supports BDNF/TrkB involvement. Following unilateral moderate sound exposure in rats, BDNF protein levels were significantly upregulated in fusiform cells of the dorsal cochlear nucleus—a structure critically involved in tinnitus generation—with a more pronounced increase observed in aged animals (Wang et al., 2011). TrkB protein levels were similarly increased in sound-exposed fusiform cells (Wang et al., 2011). The reciprocal expression patterns of BDNF and the activity-dependent plasticity marker Arg3.1 further link this pathway to tinnitus perception: as acoustic trauma increased BDNF exon IV expression in spiral ganglion neurons, Arg3.1 expression was concurrently reduced in the AC; these reciprocal changes correlated with behavioral evidence of tinnitus (Tan et al., 2007). Additionally, noise exposure and cochlear ablation upregulate BDNF expression in the inferior colliculus alongside GAD65 (glutamate decarboxylase), suggesting that BDNF participates in the excitatory/inhibitory balance within central auditory structures following acoustic insult (Erol et al., 2012). Figure 1B summarizes the neurotrophic pathways affected in tinnitus.

### 2.3 Synaptic dysfunction and abnormal neural plasticity

A core feature of neurodegenerative diseases is synaptic loss and dysfunction, a principle that finds a compelling parallel in the pathophysiology of tinnitus. Preclinical studies consistently demonstrate that tinnitus models exhibit increased spontaneous excitatory postsynaptic currents (sEPSCs) and decreased spontaneous inhibitory postsynaptic currents (sIPSCs) in pyramidal neurons of the primary AC, changes that directly correlate with behavioral evidence of tinnitus (Ghimire et al., 2023). This E/I imbalance is driven in part by aberrant activation of NMDARs, and NMDAR antagonists have shown therapeutic potential in tinnitus (Rafe et al., 2024; Zhang et al., 2026). Systematic phosphoproteomic analyses further reveal that tinnitus development critically depends on phosphorylation-mediated post-translational modifications that reshape synaptic function, particularly affecting membrane receptors and synaptic proteins (Zhang et al., 2025). These synaptic changes are not confined to the AC; they also involve somatosensory inputs to the dorsal cochlear nucleus, promoting enhanced excitability in cervicogenic somatic tinnitus (Wadhwa et al., 2024).

The immediate consequence of this E/I imbalance is neuronal hyperexcitability—an increase in spontaneous firing rates (SFRs) and bursting activity in AC neurons, which directly correlates with tinnitus behavior (Cai et al., 2024). Initially, such hyperexcitability may arise as a compensatory response to peripheral deafferentation. Following auditory deprivation, when rapid auditory processing and tonic inhibition intensity are preserved, increased central neural gain is widely observed as a compensatory mechanism that maintains hearing function (Knipper et al., 2021). However, the relationship between tinnitus and central gain remains debated (Sedley, 2019; Knipper et al., 2021). When both rapid auditory processing and tonic inhibition are impaired, some studies have reported a paradoxical decrease in central gain in certain patient subgroups, particularly those without comorbid hyperacusis (Chen et al., 2021; Wolpert et al., 2020). Conversely, other evidence—including meta-analyses and animal models—supports increased central gain in tinnitus (Chen et al., 2021; Möhrle et al., 2019). For instance, a meta-analysis of ABR studies in normal-hearing tinnitus patients found consistently elevated V/I ratios indicative of central gain enhancement, although inconsistencies in wave V amplitude changes suggest that gain alterations may be necessary for tinnitus generation but not for its maintenance (Chen et al., 2021). Wolpert et al. (2020) further demonstrated that tinnitus with hyperacusis is associated with increased central gain, whereas tinnitus without hyperacusis shows reduced gain, suggesting that hyperacusis status is a critical determinant of gain direction. These discrepancies underscore that central gain changes in tinnitus are not unidirectional but may depend on factors such as comorbidities (e.g., hyperacusis), insult type, and the specific neural measures employed (Sedley, 2019; Möhrle et al., 2019). Thus, while compensatory central gain increases are well-established in hearing loss, their specific contribution to tinnitus—whether increased, decreased, or absent—remains an open question requiring further investigation with well-controlled, hearing-matched designs (Sedley, 2019; Möhrle et al., 2019). Hyperexcitability is not limited to the auditory pathway; it extends to limbic regions and can be modulated by stress, which decreases GABA A receptor α1 expression in the hippocampus (Kim et al., 2021).

A second, related phenomenon is increased neuronal synchrony—the temporal correlation of firing across neurons. While hyperexcitability refers to firing rate, synchrony refers to coordinated activity. In tinnitus, abnormal synaptic plasticity leads to enhanced synchronous discharge among neurons in the AC and beyond, cementing the formation of a persistent “tinnitus network” (Kapolowicz and Thompson, 2019). Human neuroimaging studies reveal altered white matter integrity and functional connectivity involving not only auditory structures like Heschl’s gyrus but also the internal capsule, corpus callosum, amygdala, and hippocampus (Khan et al., 2021; Shen et al., 2025). Resting-state fMRI consistently shows abnormal spontaneous activity and increased functional connectivity between the auditory network and limbic regions (e.g., cerebellum, amygdala), with connectivity strength correlating with tinnitus severity (Cai et al., 2019). Oscillatory dynamics are also disrupted: impaired gamma phase coherence is observed in the prefrontal cortex following hearing loss (Hayes et al., 2024), and the tonotopic organization of the AC becomes disorganized, particularly for low-intensity tones (Wake et al., 2024). Large-scale human fMRI studies suggest that extensive tonotopic map reorganization is more characteristic of hearing loss itself, whereas tinnitus may involve an incomplete or more conservative form of central compensation (Koops et al., 2020). Computational models of homeostatic plasticity successfully generate neural signatures of both hyperactivity and increased neural noise, bridging microscale synaptic changes to macroscale network phenomena (Schultheiβ et al., 2023). Network pathology is also state-dependent: local cortical hyperactivity may interfere with sleep-related sensory disconnection, potentially contributing to tinnitus persistence (Milinski et al., 2022). The E/I imbalance and resulting pathology are depicted in Figure 1C.

### 2.4 Oxidative stress

Reactive oxygen species (ROS) overproduction is a pivotal shared cellular injury mechanism implicated in both tinnitus and neurodegenerative diseases (Zhang et al., 2022; Teraoka et al., 2024; Fetoni et al., 2019). In the auditory system, cochlear hair cell damage, hyperactivity of auditory neurons, and chronic neuroinflammation converge to disrupt mitochondrial function, leading to excessive ROS generation and oxidative stress (Zhang et al., 2022; Fetoni et al., 2019; Xu and Yang, 2025). Mitochondria, as the primary energy producers and regulators of redox homeostasis, become dysfunctional under these pathological conditions, resulting in an imbalance favoring ROS accumulation (Xu and Yang, 2025; He H. et al., 2025). This oxidative milieu attacks cellular macromolecules including lipids, proteins, and DNA, compromising neuronal membrane integrity and ion channel function, notably potassium channels critical for auditory signal transduction (Teraoka et al., 2024; Fetoni et al., 2019). Such molecular damage impairs the survival and function of neurons within the auditory pathway, exacerbating tinnitus symptoms (Zhang et al., 2022; He H. et al., 2025). Moreover, oxidative stress facilitates the abnormal aggregation and toxicity of neurodegenerative hallmark proteins such as *β*-amyloid (Aβ) and *α*-synuclein, linking tinnitus pathophysiology to broader neurodegenerative processes (Boutté et al., 2021). Empirical evidence supports this connection; elevated oxidative damage markers like 8-hydroxy-2′-deoxyguanosine (8-OHdG) and 4-hydroxynonenal (4-HNE) have been detected in the auditory centers and hippocampus of tinnitus patients and animal models, indicating systemic oxidative injury (Scuto et al., 2020; Fetoni et al., 2019; Boutté et al., 2021). For instance, studies in AD models demonstrate that Aβ deposition induces oxidative stress and neuroinflammation, which parallels mechanisms observed in tinnitus-related cochlear and central auditory damage (Xu et al., 2023). Similarly, in Meniere‘s disease, a disorder often accompanied by tinnitus, increased systemic oxidative stress markers and neuroinflammatory responses have been documented, underscoring the role of ROS in inner ear pathology (Scuto et al., 2020). The convergence of mitochondrial dysfunction, ROS burst, and neuroinflammation creates a vicious cycle that not only damages auditory neurons but also promotes neurodegenerative proteinopathies, suggesting that oxidative stress is a fundamental pathological nexus in tinnitus and neurodegenerative diseases (Xu and Yang, 2025; He H. et al., 2025; Boutté et al., 2021). Understanding this shared mechanism offers a rationale for therapeutic strategies targeting mitochondrial health and oxidative stress mitigation to alleviate tinnitus and potentially slow neurodegenerative progression (Teraoka et al., 2024; Fetoni et al., 2019).

The nuclear factor erythroid 2-related factor 2 (Nrf2) and antioxidant response element (ARE) signaling pathway is central to cellular defense against oxidative stress, orchestrating the transcription of phase II detoxifying enzymes and antioxidant proteins (Xu et al., 2023; Yang et al., 2025). In chronic tinnitus and neurodegenerative diseases, dysfunction of the Nrf2/ARE pathway has been increasingly recognized as a critical factor contributing to the collapse of endogenous antioxidant defenses (Scuto et al., 2020; Yang et al., 2025). Under physiological conditions, Nrf2 translocates from the cytoplasm to the nucleus upon oxidative challenge, binding to ARE sequences to induce expression of protective genes such as heme oxygenase-1 (HO-1), NAD(P)H quinone oxidoreductase 1 (NQO1), and glutamate-cysteine ligase catalytic subunit (GCLC) (Xu et al., 2023; Yang et al., 2025). However, in pathological states associated with tinnitus and neurodegeneration, this translocation is impaired or the binding affinity of Nrf2 to ARE is diminished, resulting in insufficient upregulation of antioxidant enzymes (Scuto et al., 2020; Yang et al., 2025). Consequently, cells fail to adequately detoxify accumulated ROS and electrophilic toxins, leading to oxidative damage and neuronal vulnerability (Fetoni et al., 2019; Yang et al., 2025). Experimental evidence supports this impairment; for example, hawthorn leaf flavonoids have been shown to exert neuroprotective effects in an AD model by activating the Nrf2/ARE pathway, enhancing antioxidant enzyme activities, and reducing oxidative stress and neuroinflammation (Xu et al., 2023). Similarly, in Meniere‘s disease patients, supplementation with Coriolus versicolor biomass induced vitagenes including HO-1 and other Nrf2-regulated proteins, improving systemic antioxidant capacity and mitigating oxidative damage (Scuto et al., 2020). These findings highlight the therapeutic potential of pharmacological or natural compounds that activate Nrf2 signaling to restore antioxidant defenses (Scuto et al., 2020; Xu et al., 2023; Yang et al., 2025). By enhancing endogenous antioxidative capacity, such interventions may counteract the aberrant neuroplasticity and protein aggregation underlying tinnitus and neurodegenerative diseases (Scuto et al., 2020; Boutté et al., 2021). Therefore, targeting the Nrf2/ARE pathway represents a promising strategy to simultaneously address oxidative stress-induced neuronal dysfunction and the toxic proteinopathies characteristic of these disorders, offering hope for more effective treatments that modify disease progression rather than merely alleviating symptoms (Yang et al., 2025; Scuto et al., 2020; Xu et al., 2023). Figure 1D illustrates the oxidative stress–mitochondrial dysfunction axis.

### 2.5 Epigenetic dysregulation

Beyond genetic susceptibility, emerging evidence implicates epigenetic mechanisms—heritable and reversible modifications that regulate gene expression without altering DNA sequence—in the pathophysiology of tinnitus. These mechanisms include DNA methylation, histone acetylation, and microRNA (miRNA)-mediated post-transcriptional regulation. Chronic stress, noise exposure, and neuroinflammation can induce lasting epigenetic alterations that may perpetuate maladaptive plasticity within auditory and limbic circuits, mirroring epigenetic contributions to neurodegenerative diseases such as Alzheimer’s and Huntington’s disease (Lopez-Escamez et al., 2016). Histone acetylation alterations. Histone acetylation, mediated by histone acetyltransferases (HATs) and deacetylases (HDACs), controls chromatin accessibility and transcriptional activity. In neurodegenerative disorders, pathological HDAC overactivity has been documented, leading to transcriptional silencing of neuroprotective genes (Saha and Pahan, 2006; Penney and Tsai, 2014). HDAC2, which is enriched in neurons and involved in synaptic plasticity, has been implicated in reversing maladaptive plasticity in neurological conditions. Specifically, HDAC2, which is enriched in neurons and involved in synaptic plasticity, has been implicated in reversing maladaptive plasticity in other neurological conditions (Denk et al., 2013). Moreover, reduced sirtuin (class III HDAC) activity has been observed in Meniere’s disease, a disorder frequently accompanied by tinnitus, correlating with increased oxidative stress and neuroinflammation (Scuto et al., 2020). While direct evidence in human tinnitus tissue is scarce and no study has yet demonstrated HDAC isoform alterations specifically in the AC in tinnitus models, pharmacological inhibition of HDAC3 in the AC has been shown to accelerate learning and strengthen cortical encoding of behaviorally relevant sounds, demonstrating that HDAC-mediated mechanisms can directly regulate AC plasticity (Ateşyakar and Bieszczad, 2026).

#### 2.5.1 DNA methylation changes

DNA methylation at CpG islands in promoter regions typically represses gene transcription. In Meniere’s disease, whole-genome DNA methylation profiling of peripheral blood mononuclear cells has identified distinct immune subgroups, with pathways related to cytokine stimulation and NMDA receptor signaling showing prominent differential methylation patterns (Flook et al., 2021). Beyond Meniere’s disease, saliva-based DNA methylation analysis has been used to study epigenetic changes in tinnitus patients, providing a non-invasive avenue for biomarker discovery (Bhatt et al., 2024). Furthermore, blast exposure, a common cause of acoustic trauma and tinnitus, has been associated with altered DNA methylation patterns in peripheral blood, supporting the concept that environmental insults induce lasting epigenetic footprints relevant to tinnitus pathophysiology (Wang et al., 2020). The BDNF gene is known to be regulated by DNA methylation; stress-induced changes in BDNF promoter methylation have been linked to reduced BDNF expression in animal models of chronic stress, and similar mechanisms could underlie the reduced serum BDNF levels observed in tinnitus patients (Basner et al., 2017).

#### 2.5.2 MicroRNA involvement

MicroRNAs (miRNAs) are small non-coding RNAs that post-transcriptionally silence target mRNAs. A systematic review has summarized the diverse functions of miRNAs in the auditory system, including their roles in hair cell development, survival, and responses to acoustic trauma, positioning miRNAs as key regulators of auditory homeostasis and potential contributors to tinnitus (Ebbers et al., 2022). In salicylate-induced tinnitus models, microRNA-375-3p has been shown to target ELAVL4, which subsequently modulates NR2B expression and ROS production (Zhu et al., 2024). This identifies miRNAs as upstream regulators of excitotoxicity and oxidative stress. Other miRNAs implicated in neuroinflammation (e.g., miR-146a, miR-155) and synaptic plasticity (e.g., miR-132, miR-134) warrant systematic investigation in tinnitus models. Altered miRNA profiles have been reported in the cochlea following acoustic trauma, and in tinnitus models induced by other etiologies such as salicylate, suggesting that miRNA dysregulation may contribute to maladaptive responses to peripheral injury (Patel et al., 2013). Epigenetic modifications are shown in Figure 1E.

## 3 Therapeutic strategies targeting neuroinflammation

### 3.1 Microglial modulators

The application of microglial modulators, such as the inhibitor minocycline, represents a promising therapeutic strategy for tinnitus, drawing mechanistic parallels from research in other neurodegenerative and neuroinflammatory conditions (Singh et al., 2021). This action is consistent with its known mechanism as a tetracycline derivative that suppresses activated microglia, the resident immune cells of the central nervous system (Tikka et al., 2001). Chronic neuroinflammation, characterized by sustained microglial activation and cytokine release, is a hypothesized contributor to the progression of various neurological deficits, including those following traumatic brain injury which can manifest as tinnitus (Witcher et al., 2021). Neuroinflammation is a key component of secondary injury following traumatic brain injury, and anti-inflammatory therapies have been investigated for its regulation (Izzy et al., 2025). This convergence of evidence underscores a shared pathophysiological thread where dysregulated microglial activity drives neural circuit dysfunction, making its pharmacological inhibition a rational cross-disease therapeutic approach.

Beyond broad-spectrum inhibition, the development of novel, specific microglial phenotype-modulating drugs offers a new direction for precision control of tinnitus-related inflammation. Neuroinflammation involves the participation of microglia, and multiple anti-inflammatory therapies have been explored for its regulation after traumatic brain injury (Kalra et al., 2022; El Baassiri et al., 2024; Zamanian et al., 2022; Wang S. et al., 2024). This shift is crucial because microglia play dual roles while chronic M1 activation promotes damage through cytokine release and oxidative stress, M2 activation supports tissue repair and resolution of inflammation (Tang and Le, 2016). Promoting this phenotypic conversion could theoretically resolve the maladaptive neuroinflammatory environment implicated in tinnitus while preserving the beneficial, homeostatic functions of microglia.

However, the clinical translation of microglial-targeted therapies for tinnitus faces significant challenges, primarily concerning drug delivery and long-term safety. The development of effective anti-inflammatory therapies for neuroinflammation after traumatic brain injury remains challenging (Lozano et al., 2015). The blood–brain barrier (BBB), whose permeability can be altered following injury or inflammation, poses a significant but variable obstacle to systemic drug administration (Candelario-Jalil et al., 2022). Furthermore, the long-term consequences of suppressing or modulating neuroinflammation require careful assessment. Anti-inflammatory therapies are under investigation for the regulation of neuroinflammation after traumatic brain injury (Harrison et al., 2014). The development of effective anti-inflammatory therapies for traumatic brain injury faces multiple unresolved challenges (Aarabi and Simard, 2009). Therefore, successful translation will depend on advances in targeted CNS delivery systems and a thorough understanding of the temporal dynamics of microglial activation in tinnitus pathogenesis.

### 3.2 Cytokine and inflammatory pathway antagonists

#### 3.2.1 Monoclonal antibodies targeting specific cytokines (e.g., anti-TNF-*α*) or receptor antagonists

The exploration of cytokine-targeted therapies, specifically monoclonal antibodies (mAbs) or receptor antagonists, for tinnitus represents a promising translational avenue from autoimmune and inflammatory disorders. TNF-*α* is a key pro-inflammatory cytokine implicated in neuroinflammatory processes that may underlie tinnitus induced by noise and drug (Wang et al., 2019; Mennink et al., 2022; Hwang et al., 2011; Szczepek et al., 2014). Preclinical evidence strongly suggests a pathogenic role for TNF-*α*. In a mouse model, central infusion of recombinant TNF-*α*, when combined with sub-threshold noise exposure, synergistically promoted microglial activation, loss of parvalbumin-positive inhibitory neurons in the AC, and induced auditory processing deficits and tinnitus-like behaviors, which were not observed with either insult alone (Deng et al., 2020b). This indicates that elevated brain proinflammatory cytokine levels, as might occur in systemic inflammatory conditions, can act as a risk factor, lowering the threshold for noise-induced auditory dysfunction and tinnitus. Furthermore, studies in salicylate-induced tinnitus models show significant upregulation of pro-inflammatory cytokines, including IL-1β, alongside activation of microglia and astrocytes in central auditory structures like the primary AC and medial geniculate body (Xia et al., 2020). While anti-TNF-*α* agents like infliximab or adalimumab are mainstays in treating rheumatoid arthritis, their direct application in tinnitus remains in the preclinical investigative phase. The rationale is grounded in the observed inflammatory signatures in tinnitus models and certain associated conditions. For instance, in Meniere’s disease, a condition characterized by tinnitus, vertigo, and hearing loss, cytokines like TNF-*α* and IL-1β are considered central players in defining an autoinflammatory endophenotype, suggesting a subset of patients might benefit from cytokine blockade (Frejo and Lopez-Escamez, 2022). Similarly, immune profiling in MD has identified patient clusters with a type 2 allergic inflammatory response, hinting at the potential utility of personalized interleukin-4 (IL-4) blockers (Flook et al., 2023). However, human studies directly linking specific cytokine levels to idiopathic tinnitus have yielded mixed results, with some showing alterations in cytokines like IL-10 and IFN-*γ* (Mennink et al., 2024)and others finding correlations between tinnitus intensity and IL-6 or TNF-*α* levels (Tanaka et al., 2024), while yet another study in older adults found no broad cytokine differences except for IL-10 in males (Tanaka et al., 2024). This heterogeneity underscores that, unlike the clear autoimmune pathogenesis in rheumatoid arthritis, the inflammatory component in tinnitus is likely complex and heterogeneous. Therefore, while the preclinical foundation for exploring anti-cytokine therapies exists, significant work is required to bridge this to clinical application, including identifying which tinnitus patients exhibit a dominant cytokine-driven pathology that would be amenable to such targeted biologics see in Figure 2 ⑥ and ⑦.

#### 3.2.2 Inflammatory signaling pathways such as nuclear factor kappa B (NF-κB) or p38 mitogen-activated protein kinase (MAPK)

Targeting upstream master regulators of inflammation, such as the NF-κB and p38 MAPK pathways, offers a strategic therapeutic approach by potentially suppressing a broad spectrum of downstream pro-inflammatory effectors implicated in tinnitus pathogenesis. The NF-κB pathway is a pivotal transcription factor that controls the expression of numerous genes involved in immune and inflammatory responses, including cytokines like TNF-*α*, IL-1β, and IL-6. Its activation is a common feature in neuroinflammatory states. In the context of tinnitus, evidence points to the involvement of this pathway. For example, in Meniere’s disease, an allelic variant associated with the condition is linked to NF-κB-mediated inflammation, positioning this pathway as a key mechanistic player (Frejo and Lopez-Escamez, 2022). Furthermore, DNA methylation profiling analysis of peripheral blood mononuclear cells from MD patients has defined immune subgroups, with pathways related to cytokine stimulus showing prominent differentiation in methylation patterns (Patil et al., 2025). Inhibition of this pathway could therefore dampen the production of multiple cytokines that contribute to cochlear and central auditory inflammation. Similarly, the p38 MAPK pathway is activated by cellular stress and inflammatory signals and plays a crucial role in the synthesis and release of pro-inflammatory cytokines. While direct preclinical studies on p38 MAPK inhibition in tinnitus models are not detailed in the provided references, the principle is supported by the broader neuroinflammation literature. The activation of glial cells (microglia and astrocytes) in tinnitus models is a source of these inflammatory mediators (Xia et al., 2020). Inhibiting upstream signals like p38 MAPK could prevent glial activation and the subsequent cytokine cascade. The preventive potential of such interventions is highlighted by the study on TNF-*α* and noise exposure, where the co-presence of a pro-inflammatory state and an acoustic trigger was necessary to induce auditory pathology (Deng et al., 2020b). This suggests that prophylactic or early intervention with anti-inflammatory agents that block upstream pathways like NF-κB or p38 MAPK following a known insult (noise trauma) could potentially decouple the inflammatory response from the triggering event, preventing maladaptive neural plasticity and the development of tinnitus. This approach is conceptually supported by research in other neurological conditions where neuroinflammation is a contributor, such as traumatic brain injury, where modulating the neuroinflammatory response is a therapeutic target to prevent secondary and tertiary neuronal damage (Jacquens et al., 2022). Beyond TBI, accumulating evidence from diverse neurological disorders including AD, PD and MS validates the therapeutic potential of upstream inflammatory pathway blockade (Hou et al., 2024; Iba et al., 2023). Therefore, pharmacological agents that inhibit NF-κB or p38 MAPK represent a promising class of drugs for repurposing in tinnitus, particularly for prevention in high-risk scenarios or early intervention in acute cases, by broadly suppressing the inflammatory milieu that facilitates the establishment of tinnitus see in Figure 2 ④, ⑤ and ⑧.

Preclinical studies have provided evidence that inhibiting excessive neuroimmune responses can disrupt the vicious cycle of tinnitus generation and maintenance (Jiang et al., 2025). The underlying mechanisms involve maladaptive auditory-limbic network connectivity and dysregulation of neuro-immune interactions, where astrocytes play a significant role in both neural responses to inflammation and the plasticity regulation implicated in tinnitus pathophysiology (Perin and Pizzala, 2024). The translational promise of this approach lies in the development of next-generation anti-inflammatory agents with greater central nervous system specificity and improved safety profiles. For instance, targeting specific receptors abundantly expressed in auditory thalamocortical circuits, such as muscarinic receptors, has been proposed as a potential therapeutic strategy for hearing loss and tinnitus (Sharma et al., 2025). More directly, genetic evidence from Mendelian Randomization studies has identified a causal relationship between elevated levels of specific circulating inflammatory proteins (e.g., CCL19, CXCL11, TNFSF12) and an increased risk of tinnitus. This paves the way for developing precision anti-inflammatory therapies and, coupled with advanced delivery systems like nanocarriers for targeted brain delivery, holds significant potential for providing causative treatment for tinnitus patients identified as belonging to an “inflammatory subtype” based on their specific biomolecular profile (He S. et al., 2025).

## 4 Neurotrophic factors and neuroprotective strategies

### 4.1 BDNF/TrkB signaling pathway

The therapeutic relevance of the BDNF/TrkB pathway is further supported by TrkB agonist studies in noise-induced hearing loss models, which are highly relevant to tinnitus. Pharmacological activation of TrkB with agonists such as amitriptyline or 7,8-dihydroxyflavone, administered directly into the cochlea 48 h after noise exposure, partially restored inner hair cell afferent synapse counts and auditory brainstem response wave I amplitudes, with effects persisting for up to 2 weeks (Fernandez et al., 2021). Systemically delivered amitriptyline produced even more durable effects, maintaining synaptic integrity in treated animals for up to 1 year post-treatment (Fernandez et al., 2021), underscoring the translational potential of TrkB-based strategies for auditory synaptopathy, a key pathological substrate of tinnitus.

Beyond preclinical models, human genetic studies have established a direct link between BDNF signaling and tinnitus susceptibility. The BDNF Val66Met polymorphism (rs6265) has been shown to regulate vulnerability to chronic stress and phantom perception, with Val/Met carriers exhibiting increased alpha power in the subgenual anterior cingulate cortex that correlates with distress levels; distress mediates the relationship between BDNF Val66Met polymorphism and tinnitus loudness (Vanneste et al., 2021). Furthermore, polymorphisms in the BDNF antisense (BDNF-AS) gene—specifically rs925946, rs1519480, and rs10767658—show significant associations with chronic tinnitus, with rs10767658 conferring a 2.33-fold increased risk in the recessive model and demonstrating associations with tinnitus duration (Yuksel et al., 2023). Reduced serum BDNF levels have also been observed in tinnitus patients compared to controls, further supporting a role for this neurotrophic pathway in tinnitus pathophysiology (Coskunoglu et al., 2017).

Given the neuroprotective potential of intact BDNF/TrkB signaling, therapeutic strategies aimed at augmenting this pathway are of significant interest, mirroring approaches explored in other neurodegenerative contexts like Huntington’s disease (Couly et al., 2021). Pharmacological agents that directly activate TrkB, such as the flavonoid 7,8-dihydroxyflavone (7,8-DHF), have demonstrated promise in preclinical models. Natural flavones like quercetin and apigenin act as TrkB agonists, directly binding to the receptor’s extracellular domain to activate the downstream CREB pathway, thereby reducing apoptosis, oxidative stress, and protein aggregation in cellular models of neurodegeneration (Chiu et al., 2023). Beyond direct receptor agonism and cochlear BDNF delivery, non-pharmacological interventions such as physical exercise and environmental enrichment represent potent modulators of the endogenous BDNF/TrkB system. Aerobic exercise can increase serum BDNF and TrkB levels in male football players (Jahani et al., 2025). Similarly, environmental enrichment, which provides enhanced sensory, cognitive, and motor stimulation, can elevate BDNF levels and, when combined with therapies that bolster TrkB expression (as demonstrated with the peptide P42 in an HD mouse model), synergistically delays disease progression and improves motor and cognitive outcomes (Couly et al., 2021). These findings underscore the potential of both pharmacological TrkB agonists and lifestyle interventions to enhance BDNF/TrkB signaling, offering a multi-modal strategy to counteract the synaptic dysfunction and neuronal vulnerability that may underlie tinnitus see in Figure 2 ⑨ and ⑩.

However, the therapeutic targeting of the BDNF/TrkB pathway is fraught with significant challenges, primarily centered on the need for precise spatiotemporal control to avoid the detrimental effects of pathway overactivation or imbalance. Excessive or sustained activation of downstream effectors like ERK1/2, while crucial for plasticity, can itself contribute to neurodegeneration under pathological conditions (Numakawa and Kajihara, 2026). BDNF/TrkB signaling is involved in the occurrence, development, growth and metastasis of multiple cancers including brain, breast, urological, and gastrointestinal cancers (Guzel et al., 2021). A major translational hurdle is the development of agents that can effectively cross the BBB to reach central auditory structures. Some small molecule TrkB agonists like LMDS-1 and LMDS-2 have shown BBB permeability in parallel artificial membrane permeability assays (Chiu et al., 2022). Another layer of complexity involves the precise regulation of TrkB receptor trafficking and signaling termination. For instance, the cysteine protease legumain can cleave TrkB, potentially generating soluble receptors that act as BDNF scavengers, thereby attenuating signaling—a mechanism that may be relevant in neurodegeneration (Holzner et al., 2023). Additionally, the small GTPase Rab10 is critical for sorting internalized TrkB into signaling endosomes for retrograde transport, a process essential for propagating neurotrophic signals from synapses to the cell body; dysregulation here could impair neuroprotective signaling (Lazo and Schiavo, 2023). In acute ischemic stroke, TrkB mediates pro-survival signaling while p75NTR mediates pro-apoptotic signaling, and the therapeutic strategy involves activating TrkB signaling and inhibiting p75NTR signaling (Alnoaman et al., 2025).

### 4.2 Potential of other neurotrophic factors

Glial cell line-derived neurotrophic factor (GDNF) is a potent neurotrophic factor with a wide range of biological actions that positively affect the viability, proliferative activity, and migratory ability of cells in the nervous system (Revishchin et al., 2022). It is a highly conserved protein critical for organismal development and survival, known for promoting the survival and differentiation of dopaminergic neurons and preventing apoptosis in mature neurons (Shamadykova et al., 2025; Cintrón-Colón et al., 2020). These properties make GDNF a prominent candidate for therapeutic applications in neurodegenerative diseases, particularly PD, where it has been extensively researched for its neuroprotective effects on the dopaminergic nigrostriatal pathway (Kakoty et al., 2024; Kambey et al., 2021). The neuroprotective mechanism involves GDNF binding to its co-receptor GFRα1 and the receptor tyrosine kinase RET, activating downstream signaling cascades such as PI3K/AKT and ERK1/2, which promote neuronal survival and function (Khan et al., 2025). GDNF has been investigated preclinically and clinically for alleviating PD-related symptoms (Kakoty et al., 2024). Upregulation of endogenous GDNF does not provide dopaminergic neuroprotection in a proteasome inhibition mouse model of PD (Olfat et al., 2023). For instance, while ultrasound-targeted microbubble destruction (UTMD) systems for GDNF or BDNF gene delivery show synergistic neuroprotective effects in PD models, simultaneous GDNF/BDNF delivery did not show additional benefits, possibly due to competing expressions (Lin et al., 2020). Furthermore, the biological activity and neuroinductive properties of GDNF are highly dependent on its production system and post-translational modifications, with mammalian cell-produced GDNF showing superior efficacy compared to bacterial systems (Shamadykova et al., 2021).

Recent strategies to overcome these hurdles include the development of novel delivery systems such as gene-nanocarrier complexes, the use of human induced pluripotent stem cell-derived neural progenitor cells secreting GDNF (iNPC-GDNFs) for combined cell and gene therapy, and the exploration of small neurotrophic peptides like djGDNF47 generated via alternative splicing (Shamadykova et al., 2025; Lin et al., 2020; Laperle et al., 2023). GDNF promotes the survival and differentiation of dopaminergic neurons (Shamadykova et al., 2025). In epilepsy research, GDNF-releasing mesenchymal stem cells can modulate epileptogenesis (Waloschková et al., 2025). However, GDNF’s pleiotropic effects also present a double-edged sword, as its overexpression can promote the growth and therapy resistance of high-grade gliomas, underscoring the need for precise, targeted therapeutic strategies (Revishchin et al., 2022). In the context of tinnitus, bioinformatic studies of synaptic transmission in spiral ganglion neurons have identified GDNF as a key protein, with protein–protein interaction networks highlighting its role alongside BDNF in processes related to the perception of sound (Gross et al., 2023). While genetic association studies for common variants in the GDNF gene have been underpowered for tinnitus, plasma biomarker studies have identified GDNF among top circulating proteins associated with constant tinnitus, though these associations did not survive strict multiple testing corrections (Amanat et al., 2021; Cederroth et al., 2023). GDNF has a strong neuroprotective profile and is being actively explored for the treatment of neurodegenerative diseases, particularly gene therapy approaches for PD (Kakoty et al., 2024; Khan et al., 2025).

Nerve growth factor (NGF), the first identified neurotrophic factor, plays a crucial role in the growth, differentiation, and survival of neurons in both the peripheral and central nervous systems (Norata et al., 2024). NGF exerts its biological effects by binding to its high-affinity tropomyosin receptor kinase A (TrkA), which activates pro-survival pathways including PI3K/AKT and MAPK/ERK, and its low-affinity p75 neurotrophin receptor (p75NTR), which can mediate pro-apoptotic signals (Rogdakis et al., 2022). NGF has demonstrated significant neuroprotective potential across various neurological disorders, including AD, PD, retinal degenerative diseases, and conditions involving nerve injury (Norata et al., 2024; Esposito et al., 2021). NGF is a promising therapeutic target to slow or prevent neurodegenerative progression in AD (Rogdakis et al., 2022; Manni et al., 2021). Similarly, in ocular disorders like diabetic retinopathy, age-related macular degeneration and some vitreoretinal diseases, NGF exerts neuroprotective activity on retinal ganglion cells (RGCs) and can promote whole retina restoration (Esposito et al., 2021). However, the clinical translation of NGF protein therapy faces substantial challenges. NGF has poor bioavailability and cannot efficiently cross the BBB (Rogdakis et al., 2022), and it also has a short half-life (Jiang et al., 2024). To overcome these barriers, innovative delivery strategies are being actively pursued. These include intranasal administration, which allows NGF to bypass the BBB and reach brain parenchyma, and the development of NGF-mimetic small molecules or selective TrkA agonists (Rogdakis et al., 2022; Manni et al., 2021). For example, lipid nanoparticle-formulated circular RNA expressing NGF (circNGF) provides sustained protein expression and robust neuroprotection for RGCs, significantly outperforming NGF protein therapy (Jiang et al., 2024). Other strategies involve ROS-responsive ruthenium nanoplatforms, amphiphilic solid lipid nanoparticles functionalized with targeting peptides, and magnetic field-stimulated superparamagnetic nanoparticles to deliver NGF and enhance neuronal differentiation (Yuan et al., 2021; Kuo et al., 2021; Georgas et al., 2023).

The therapeutic potential of NGF extends beyond pure neuroprotection to modulating emotional and affective states. NGF and its receptor TrkA are implicated in psychiatric disorders such as depression and schizophrenia, where altered brain levels of neurotrophins are commonly observed (Ciafrè et al., 2020). This connection is highly relevant for tinnitus, a condition frequently comorbid with anxiety (Amanat et al., 2021). Bioinformatic analyses of synaptic transmission in spiral ganglion neurons have highlighted NGF and its interaction with NTRK1 (TrkA) and NGFR (p75NTR) as potentially relevant to the tinnitus process, suggesting that the NGF-signaling pathway may be of comparable importance to BDNF signaling in tinnitus networks although this awaits functional validation (Gross et al., 2023). This suggests that an imbalance in the NGF/TrkA/p75NTR pathway may be associated not only with the aberrant auditory perception in tinnitus but also with the accompanying affective comorbidities. NGF is associated with depression and other psychiatric disorders (Ciafrè et al., 2020). Supporting this notion, studies in other neurological contexts show that compounds which potentiate NGF signaling or act as TrkA agonists can produce neuroprotective and neurite outgrowth effects, which are crucial for neural repair (Udomruk et al., 2020; Sisti et al., 2021). For instance, the flavonoid luteolin directly binds NGF and potentiates TrkA receptor signaling, and when combined with low-dose NGF, it mimics the effects of a high dose of NGF and promotes neurite outgrowth (Xiong Gao et al., 2021). Similarly, the phytochemical carvacrol induces neurite outgrowth by activating the TrkA receptor and its downstream pathways independently of NGF (Sisti et al., 2021). Furthermore, the NGF-signaling pathway is activated by crotapotin, a component of snake venom, which suggests neuroprotective effects against PD-mimetic toxins (Bernardes et al., 2024). These findings underscore the tractability of the NGF pathway for pharmacological intervention. In the specific context of hearing and tinnitus, a conceptual link exists in the proposed “brain-ear-heart axis,” where neurotrophic factor supplementation to the cochlea could inhibit pathological protein aggregation and support neuronal health, potentially preventing conditions like AD and associated amyloid cardiomyopathy (Shityakov et al., 2021). While direct evidence for NGF’s efficacy in tinnitus patients remains to be established, its established role in neuronal survival, synaptic plasticity, and emotional regulation, combined with emerging bioinformatic evidence of its involvement in tinnitus-related synaptic processes, provides a compelling rationale for further investigating the hypothesis that targeting the NGF/TrkA pathway could simultaneously ameliorate auditory perceptive distortions and the debilitating emotional symptoms that often accompany chronic tinnitus (Gross et al., 2023; Ciafrè et al., 2020). These findings provide a strong theoretical basis for developing biological agents or small-molecule agonists based on neurotrophins (Tröger et al., 2025; Abraham and Telias, 2025; Vargas et al., 2026). The clinical translation pathway may involve the safe and sustained delivery of neurotrophins to the inner ear or central auditory targets using novel drug delivery technologies, such as sustained-release hydrogels or engineered nanoparticles (Sefiani et al., 2026; Smoliński et al., 2025). The exploration of synthetic neurotrophin mimetics or peptidomimetics, which can overcome the pharmacokinetic limitations of native proteins, represents a promising direction for therapeutic development (Sefiani et al., 2026; Zota et al., 2025). Furthermore, multimodal strategies combining neurotrophic support with other regenerative approaches may enhance efficacy in treating neural damage and degeneration (Smoliński et al., 2025).

## 5 Counteracting excitotoxicity and ion channel regulation

### 5.1 NMDA receptor antagonists

Acoustic trauma causes damage to inner ear structures, and subsequent imbalance of synaptic neurotransmitters and disturbance of GABA/glutamate levels in the central auditory pathway are associated with tinnitus (Zemaitis et al., 2021). This imbalance between excitatory glutamate and inhibitory GABA is a central feature, with direct measurements in animal models showing altered GABA/glutamate homeostasis in key auditory centers like the inferior colliculus and AC following acoustic trauma (Zemaitis et al., 2021). The resulting neuronal hyperactivity is thought to underlie the phantom sound perception. This excitatory-inhibitory dysregulation is further implicated in the frequent co-occurrence of tinnitus with anxiety and sleep disorders, suggesting shared neurobiological substrates involving maladaptive auditory-limbic network connectivity and neurotransmitter imbalances (Jiang et al., 2025). The glutamatergic hypothesis is also supported by metabolomic studies in related conditions, such as hypertension with tinnitus symptoms, which identify perturbations in alanine, aspartate, and glutamate metabolism pathways (Jialiken et al., 2025). Within this framework, the NMDA subtype of glutamate receptors plays a pivotal role. Pathological conditions, such as high-dose salicylate administration, are known to increase NMDA receptor expression and function, leading to elevated calcium influx, mitochondrial dysfunction, ROS production, and excitotoxic neuronal injury, which are hallmarks of the tinnitus state (Song et al., 2021).

This excitotoxicity is not limited to cortical areas but also occurs in subcortical structures like the IC, where salicylate-induced neuronal hyperactivity and oxidative injury are closely linked to NMDA receptor-mediated mechanisms (Wang et al., 2023). Furthermore, noise-induced tinnitus models suggest that compensatory mechanisms, potentially involving nitric oxide (NO) signaling, act to increase neuronal gain in the ventral cochlear nucleus in response to reduced input, a process that appears to involve NMDA receptor facilitation (Hockley et al., 2020). The involvement of specific NMDA receptor subunits, particularly those containing the GluN2B subunit (NR2B), is strongly implicated. Salicylate exposure upregulates NR2B expression in neuronal cells, and this upregulation is associated with increased expression of immediate early genes and inflammatory markers like ARC and TNFα, contributing to the pathological cascade (Song et al., 2021). Interventions that downregulate NR2B, such as valproic acid or resveratrol, demonstrate protective effects against salicylate-induced neuronal changes and behavioral evidence of tinnitus in animal models (Song et al., 2021; Song et al., 2022). The cochlea itself is a site of NMDA receptor activity, particularly under pathological conditions. While these receptors are present at the synapse between inner hair cells and spiral ganglion neurons, their activation in the mature cochlea is often linked to excitotoxicity, with ototoxic drugs like aspirin enabling their response (Wang et al., 2021). The endogenous co-agonist D-serine has been identified as a key modulator promoting the activation of these otherwise “silent” cochlear NMDA receptors in pathological situations, presenting a novel druggable target for sensorineural hearing disease including tinnitus (Wang et al., 2021).

Given the central role of NMDA receptors in tinnitus-related excitotoxicity and synaptic plasticity, NMDA receptor antagonists have been investigated as a rationally based therapeutic strategy, repurposing knowledge from neurodegenerative conditions like AD where drugs such as memantine are used (Rafe et al., 2024). Clinical trials with various NMDA receptor antagonists have yielded mixed and inconclusive results. A recent comprehensive review notes that, while NMDA receptor antagonists have shown potential, the evidence from clinical research is mixed and insufficient to prove their general effectiveness for tinnitus (Che et al., 2025). For instance, caroverine, a non-NMDA and NMDA receptor antagonist, was found in a quasi-experimental study (a quantitative study design used to evaluate the effectiveness of an intervention when a randomized controlled trial is not ethically or practically feasible) to be superior to standard care in reducing symptoms of mild cochlear synaptic tinnitus, improving Tinnitus Handicap Inventory scores and overall symptom reduction (Dash et al., 2024; Andrade, 2021; de Vocht et al., 2021). A double-blind randomized controlled trial demonstrated that adding memantine to conventional cinnarizine treatment significantly reduced tinnitus severity index scores compared to placebo, suggesting a beneficial effect (Pourayyoubi et al., 2025). However, other controlled studies have failed to demonstrate consistent efficacy. A double-blind, randomized, placebo-controlled crossover study of memantine monotherapy (60 patients, 43 completed) found no significant improvement in Tinnitus Handicap Inventory scores compared with placebo, leading the authors to conclude that the study does not provide evidence to recommend memantine for tinnitus treatment (Figueiredo et al., 2008). The incidence of side effects during memantine treatment was 9.4%, leading to treatment interruption in all cases where they occurred. These divergent outcomes underscore the complexity of targeting NMDA receptors in tinnitus and highlight the need for more precise interventions. As research highlights, targeting specific NMDA receptor subtypes, particularly those containing the GluN2B subunit, may offer a superior therapeutic window (Rafe et al., 2024). However, despite a sound mechanistic rationale, no NMDA receptor antagonist has yet received regulatory approval for tinnitus treatment, reflecting the substantial challenges remaining in this field (Che et al., 2025).

This underscores the need for more precise interventions targeting auditory pathway-specific NMDA receptor subtypes. The heterogeneous composition of NMDA receptors, formed by different subunits (GluN1, GluN2A-D, GluN3), offers an opportunity for targeted therapy. As research highlights, targeting specific NMDA receptor subtypes could lead to medications with better clinical effects and neuroprotective properties (Rafe et al., 2024). The GluN2B subunit is a prime candidate for such selective intervention due to its specific involvement in tinnitus pathophysiology, as evidenced by its upregulation in salicylate models, where valproic acid pretreatment can attenuate this upregulation (Song et al., 2021). Subtype-selective antagonists or allosteric modulators may therefore provide a superior therapeutic window. For example, positive allosteric modulators (PAMs) that selectively enhance NMDA receptor activity specifically on GABAergic inhibitory neurons present a novel approach. *In vivo* studies show that such targeted enhancement can increase inhibitory neuron spiking, rebalance excitatory-inhibitory activity in the AC, and even prevent noise-induced tinnitus, offering a mechanism-based strategy that avoids general receptor blockade (Deng et al., 2020a). NMDA receptor antagonists depicted in Figure 2, target ①-③.

Other investigational compounds like acamprosate, neramexane, and AM-101 have also shown promise in clinical trials, though none are yet approved, highlighting the ongoing research need (Che et al., 2025). The exploration of epigenetic and molecular regulators further refines this approach. For instance, microRNA-375-3p has been shown to alleviate salicylate-induced neuronal injury *in vitro* by targeting ELAVL4 and subsequently modulating NR2B expression and ROS production, pointing to potential upstream targets for intervention (Zhu et al., 2024). Furthermore, DNA methylation studies in conditions like Meniere’s disease, which involves tinnitus, predict phenotypes related to abnormal NMDA-mediated receptor currents, suggesting epigenetic mechanisms that could influence receptor function and be targeted therapeutically (Flook et al., 2021). The complexity of the system is illustrated by findings that systemic salicylate-induced hyperactivity in the IC depends on nitric oxide signaling within that structure, which is known to mediate NMDA receptor signaling, indicating that interventions could target points in the signaling cascade upstream or downstream of the receptor itself (Olthof et al., 2022). Despite the rationale, not all evidence supports a simple blockade strategy. One experimental study in rats found that memantine did not offer substantial histological protection to cochlear structures or significantly reduce tinnitus in a salicylate model, suggesting that different mechanisms (salicylate-induced vs. noise exposure) can lead to tinnitus, and the non-selective NMDA receptor antagonist memantine does not produce significant tinnitus relief (Pavlidis et al., 2023). This reinforces the argument for precision. In summary, while glutamatergic excitotoxicity via NMDA receptors is a well-supported mechanism in tinnitus, the clinical application of broad antagonists has been limited. The future lies in developing allosteric modulators or subunit-selective antagonists (particularly for GluN2B-containing receptors) that can precisely modulate pathological hyperactivity in the auditory pathway without disrupting essential NMDA receptor functions elsewhere in the brain, thereby offering a more effective and tolerable treatment strategy for tinnitus (Rafe et al., 2024; Che et al., 2025).

### 5.2 Potassium channel openers

Potassium channel openers represent a promising class of pharmacological agents for modulating neuronal excitability, with their ability to induce membrane hyperpolarization offering a direct mechanism to counteract pathological hyperactivity, such as that observed in tinnitus. A key target is the Kv7 (KCNQ) family of voltage-gated potassium channels. Homomeric or heteromeric tetramers of Kv7.2–7.5 subunits can generate the M-current (I_M), a crucial regulator of resting membrane potential and neuronal excitability (Sun and Undem, 2024). Retigabine is a non-selective Kv7.2–7.5 activator that can inhibit the excitability of human induced pluripotent stem cell-derived sensory neurons in chronic pain research (Estacion et al., 2023). The therapeutic potential of Kv7 openers is underscored by genetic evidence; for instance, gain-of-function variants in KCNQ2 or KCNQ3 genes, which encode Kv7.2 and Kv7.3 subunits, have been associated with reduced pain sensitivity in individuals carrying a pro-excitatory Nav1.7 mutation, demonstrating how enhanced M-current can confer resilience to neuronal hyperexcitability (Estacion et al., 2023). In the context of tinnitus, noise-induced hyperactivity in the auditory pathway is linked, at least in part, to decreased Kv7.2/3 channel activity (Marinos et al., 2021). Preclinical studies using specific Kv7.2/3 activators like RL-81 have shown significant promise. In a validated operant mouse model of tinnitus, transient administration of RL-81 1 week after acoustic trauma mitigated the development of behavioral evidence of tinnitus, without affecting the underlying hearing loss (Marinos et al., 2021). This aligns with findings in human induced pluripotent stem cell-derived sensory neurons (iPSC-SNs), where retigabine and the specific Kv7.2/3 activator ICA-110381 hyperpolarized the resting membrane potential, increased action potential threshold, and robustly inhibited neuronal firing, whereas the Kv7.1-specific activator ML277 had no effect, confirming the critical role of Kv7.2/3 subunits in regulating human sensory neuron excitability (Estacion et al., 2023).

Beyond Kv7 channels, other potassium channels contribute to excitability control in auditory circuits. In the dorsal CN, cartwheel interneurons provide potent glycinergic inhibition to fusiform cells, and their spontaneous firing is dynamically regulated by ATP-sensitive potassium (K_ATP) channels (Strazza et al., 2021). Cartwheel neurons expressing open K_ATP channels remain quiescent, and pharmacological activation of these channels with diazoxide can halt spontaneous firing; diazoxide reduces glycinergic neurotransmission in the dorsal cochlear nucleus, thereby decreasing inhibition on fusiform cells, whose hyperactivity is a hallmark in animal models of tinnitus (Strazza et al., 2021). Furthermore, metabolic demands influence this system; intense neuronal activity can deplete intracellular ATP, leading to K_ATP channel opening and a consequent reduction in firing, serving as a dynamic, activity-dependent brake on excessive excitation in the auditory brainstem (de Siqueira et al., 2025). Large-conductance calcium-activated potassium (BK) channels also modulate central auditory processing. The BK channel opener BMS-191011 was shown to suppress behavioral evidence of salicylate-induced tinnitus in mice, with local application in the inferior colliculus reversing salicylate-suppressed spontaneous activity, particularly in the frequency region corresponding to the tinnitus percept, highlighting a key site of action see in Figure 2 ⑭-⑯ (Scott et al., 2022). The therapeutic induction of hyperpolarization is not limited to pharmacology.

Optogenetic tools like the recently discovered kalium channelrhodopsins (KCRs), which are natural light-gated potassium channels, enable precise, cell-specific hyperpolarization and suppression of firing, offering a powerful experimental approach to dissect and potentially correct circuit-level hyperactivity (Govorunova et al., 2022). Similarly, activation of the optogenetic chloride pump halorhodopsin induces neuronal hyperpolarization, which secondarily drives potassium ions into cells, lowering extracellular potassium concentration and indirectly inhibiting neighboring non-expressing neurons, demonstrating a network-wide inhibitory effect (Parrish et al., 2023). In summary, potassium channel openers, by stabilizing the resting membrane potential and reducing neuronal excitability, target a fundamental mechanism of pathological hyperactivity. Their efficacy in preclinical models of tinnitus, particularly through modulation of Kv7.2/3, K_ATP, and BK channels in key auditory and limbic structures, provides a compelling translational framework. The lessons from their application in epilepsy and pain, combined with emerging genetic and optogenetic evidence, strongly support their continued investigation as a viable strategy for tinnitus treatment.

## 6 Mitochondrial function and oxidative stress intervention

Mitochondrial damage, excessive ROS production, inflammatory dysregulation, and abnormal apoptosis are involved in the pathogenesis and progression of sensorineural hearing loss (SNHL) (He H. et al., 2025; Xu and Yang, 2025). The cochlea, particularly its metabolically active hair cells, spiral ganglion neurons, and the stria vascularis, is exceptionally rich in mitochondria, rendering it highly vulnerable to oxidative stress (Kishimoto-Urata et al., 2022; Okur and Djalilian, 2022). In age-related hearing loss (ARHL), bioinformatic analyses have identified key mitochondrial genes, such as Aco2, Bcs1l, and Ndufs1, which are downregulated and involved in processes like ROS metabolism and energy production, directly linking mitochondrial dysfunction to auditory decline (Ma et al., 2025). Hair cells rely on mitochondria to maintain intracellular calcium homeostasis, and mitochondria are involved in cell death pathways, with noise exposure able to cause hair cell damage (Holmgren and Sheets, 2020). This pathological ROS overproduction is a common final pathway in various forms of acquired hearing loss, including noise-induced and drug-induced (cisplatin) hearing impairment, and current evidence from human studies supporting antioxidant-based interventions remains insufficient (Teraoka et al., 2024). Mitochondrial dysfunction plays an important role in the pathogenesis of SNHL, and targeting mitochondria is a promising therapeutic strategy (Okur and Djalilian, 2022). Preclinical studies in hearing loss models support this translational potential. For instance, antioxidants have been shown to protect zebrafish lateral-line hair cells from ototoxic damage, and mitochondrial transplantation, a novel form of therapy, enhances bioenergetics and mitigates oxidative stress-induced apoptosis in auditory cells (Holmgren and Sheets, 2020; Okur et al., 2025). Furthermore, inhibiting the mitochondrial permeability transition pore (mPTP), a key mediator of mitochondrial-driven cell death, with compounds like DBP-iPT has shown promise in protecting against cisplatin-induced ototoxicity by preserving mitochondrial membrane potential and dynamics (Kim Y. R. et al., 2025).

To achieve more comprehensive and sustained cytoprotection, a synergistic strategy combining mitochondria-targeted antioxidants with agents that promote mitochondrial biogenesis and quality control is emerging. For example, the mitophagy inducer Urolithin A prevented ARHL in mice by enhancing the clearance of damaged mitochondria and improving mitochondrial DNA integrity and ATP production in the auditory system (Cho et al., 2024). Similarly, drugs like atorvastatin have been repurposed to delay ARHL progression by activating pathways such as HSF1/Sirt1, which bolster antioxidant defenses and maintain mitochondrial structure (Choo et al., 2022). Peroxisome proliferator-activated receptor-gamma (PPAR-*γ*) agonists are another class of compounds known to enhance mitochondrial biogenesis and function. Antioxidant delivery and promotion of mitochondrial biogenesis are both key mitochondria-targeted therapeutic strategies for SNHL (He H. et al., 2025). This combined approach addresses both the acute oxidative insult and the underlying metabolic capacity and resilience of auditory cells, offering a promising, multi-faceted therapeutic avenue for preventing noise-induced hearing loss and its frequent sequela, tinnitus, by safeguarding the energetic and redox homeostasis of the auditory system see in Figure 2 ⑪-⑬.

## 7 Epigenetic regulation strategies

Chronic stress and inflammation can lead to epigenetic alterations in genes related to synaptic plasticity and inflammation. In the context of neurodegenerative diseases, the exploration of therapeutic targets is a prominent theme in pharmacological research (Insel et al., 2019). While the precise molecular mechanisms of tinnitus are not fully understood, and the contribution of genetic and epigenetic factors remains an area of active investigation (Lopez-Escamez et al., 2016), parallels can be drawn from other neurological conditions. Histone deacetylase (HDAC) inhibitors, such as valproate and vorinostat, have been studied in models of neurodegenerative diseases like HD, where they are known to improve gene expression profiles and functional outcomes by modulating epigenetic states (Mielcarek et al., 2011; Pogoda et al., 2021). The underlying rationale is that chronic pathological states, including those potentially contributing to tinnitus, may involve maladaptive epigenetic silencing of genes crucial for neuronal health and plasticity. Although direct evidence specifically linking HDAC inhibitors to tinnitus mechanisms is limited, the broader principle is that agents capable of reversing detrimental epigenetic changes hold therapeutic potential for various brain disorders, including those with symptoms like tinnitus. The induction of protective cellular pathways, such as those involving vitagenes like sirtuins (a class of HDACs), has been proposed as a related neuroprotective strategy observed in other auditory pathologies like Meniere’s disease, which is characterized by tinnitus and considered a model of cochlear neurodegeneration (Scuto et al., 2020). Therefore, the foundational concept is that HDAC inhibitors could address a core pathological process—dysregulated gene expression due to epigenetic modifications—that may be shared across several neurological and auditory conditions.

The proposed mechanism of HDAC inhibitors in tinnitus—based largely on evidence from other neurological conditions—suggests that they may reverse maladaptive plasticity by increasing the expression of neurotrophic factors and suppressing pro-inflammatory cytokine production (Sada et al., 2020; Ateşyakar and Bieszczad, 2026; Durham et al., 2017; Ye et al., 2024). However, direct evidence in tinnitus models is still sparse, and this mechanism remains to be fully validated. The maladaptive neural plasticity believed to underlie chronic tinnitus could be influenced by epigenetic mechanisms. By inhibiting HDACs, these compounds promote a more permissive chromatin state, potentially enabling the transcription of genes that support synaptic resilience, reduce neuroinflammation, and restore normal neural network function (Saha and Pahan, 2006; Penney and Tsai, 2014). This epigenetic modulation has been shown to reverse maladaptive plasticity in another neurological disorder, providing a mechanistic rationale for its potential application in tinnitus (Denk et al., 2013). Furthermore, the management of oxidative stress and neuroinflammation is a critical aspect of treating neurodegenerative processes, as seen in AD models where flavonoid compounds exhibit neuroprotective effects by modulating antioxidant pathways (Xu et al., 2023). Similarly, in Meniere’s disease, interventions that induce protective vitagene pathways (which can include sirtuin activation) help counteract oxidative stress and neuroinflammation, processes also implicated in tinnitus perception (Scuto et al., 2020). Thus, HDAC inhibitors could theoretically mitigate tinnitus by targeting these convergent pathological hallmarks: aberrant plasticity and inflammation. However, a significant challenge is the non-specificity of broad-spectrum HDAC inhibitors, which can lead to off-target effects and side effects by affecting acetylation states in numerous cell types and tissues beyond the auditory pathway, limiting their clinical utility for a condition like tinnitus.

Future directions to overcome these limitations involve the development of inhibitors targeting specific HDAC isoforms (such as HDAC2) or creating auditory pathway-specific delivery systems. The goal is to enhance therapeutic precision and minimize systemic side effects. Genetic study of tinnitus is advocated as a means to identify novel molecular targets for drug development, as the contribution of genetic and epigenetic factors to tinnitus remains unclear (Lopez-Escamez et al., 2016). Focusing on specific HDAC isoforms could allow for more refined modulation of the epigenetic landscape in neurons involved in auditory processing and tinnitus generation. For instance, HDAC2 has been particularly implicated in synaptic plasticity and memory processes; its selective inhibition might more precisely reverse the maladaptive plasticity associated with tinnitus without the broader transcriptional consequences of pan-inhibitors. Additionally, targeted delivery systems, such as intra-tympanic or nanoparticle-based approaches, could direct the drug specifically to the cochlea or central auditory structures, increasing local efficacy while reducing exposure to other brain regions and peripheral organs. This strategy of targeted intervention is consistent with the broader theme of developing precision therapies in pharmacology. The rationale is supported by research in related fields; for example, nutritional interventions in Meniere’s disease aim to boost intrinsic cellular defenses in vulnerable neurons like ganglion cells (Scuto et al., 2020), a concept that could be extended to pharmacologically protecting auditory neurons via targeted epigenetic modulation. While transcranial magnetic stimulation (TMS) represents a different neuromodulatory approach for tinnitus (Wassermann and Zimmermann, 2012), the principle of focal intervention is analogous. Therefore, advancing from non-specific epigenetic modifiers to isoform-selective inhibitors or locally delivered agents represents a promising translational path to harness the potential benefits of HDAC modulation for tinnitus treatment while mitigating the risks associated with systemic, broad-action compounds.

## 8 Prospects for cell replacement and regenerative medicine

The therapeutic application of stem cells in neurodegenerative diseases, such as ALS, has increasingly focused on their paracrine actions rather than direct neuronal replacement. In these contexts, transplanted mesenchymal stem cells (MSCs) are understood to exert neuroprotective effects primarily through the secretion of a cocktail of neurotrophic factors, modulation of local immune responses, and reduction of neuroinflammation. This paradigm shift from cell replacement to trophic support and immunomodulation is critical, as it suggests that functional recovery can be achieved by improving the pathological microenvironment that contributes to neuronal dysfunction and death. The mechanisms involve the release of factors like BDNF, GDNF, and vascular endothelial growth factor (VEGF), which collectively promote neuronal survival, enhance synaptic plasticity, and suppress detrimental glial activation. This foundational principle from broader neurology provides a compelling rationale for exploring similar strategies in auditory pathologies like tinnitus, where central maladaptive plasticity and cochlear synaptopathy are key features. The hypothesis is that by mitigating inflammatory cascades and providing trophic support, stem cell-derived factors could interrupt the cycle of peripheral deafferentation and central gain increase that underlies tinnitus perception.

Translating this concept to tinnitus models, stem cell transplantation is theorized to potentially ameliorate the condition by improving the cochlear microenvironment or by providing neurotrophic support to central auditory nuclei. Tinnitus often arises from cochlear damage, such as noise-induced synaptopathy between inner hair cells and auditory nerve fibers, leading to reduced peripheral input and subsequent compensatory hyperactivity in the central auditory pathway (Tziridis et al., 2025; Diensthuber and Stöver, 2024). This synaptopathy is a key pathological substrate, and interventions that promote synaptic repair are of high interest (Tziridis et al., 2025). Stem cells, through their paracrine secretions, could target this peripheral pathology by promoting the survival of spiral ganglion neurons or the regeneration of IHC ribbon synapses, thereby restoring balanced input to the brainstem. Treatment with NAC and HPN-07 can reduce tinnitus-related neuropathy and maladaptive neural plasticity (Lu et al., 2021). However, research directly investigating stem cell transplantation for tinnitus remains in its nascent stages. Current preclinical models primarily utilize pharmacological agents or other interventions to demonstrate the principle that repairing cochlear synaptopathy can reverse behavioral evidence of tinnitus (Lu et al., 2021; Tziridis et al., 2025). The use of advanced model systems, such as inner ear organoids derived from cochlear stem/progenitor cells or pluripotent stem cells, holds promise for future basic and translational research by providing platforms to study cell-based therapies and their mechanisms (Diensthuber and Stöver, 2024). Nonetheless, direct evidence for stem cell efficacy in established tinnitus models is extremely preliminary, and most discussions remain theoretical, extrapolating from mechanisms observed in hearing loss and broader neural repair.

The path toward developing effective stem cell-based therapies for tinnitus is fraught with significant challenges. A primary hurdle is determining the optimal cell type; choices range from mesenchymal stem cells and neural stem cells to induced pluripotent stem cell (iPSC)-derived progenitors, each with distinct paracrine profiles and integration potentials. The transplantation route is another critical variable, as the choice between systemic (e.g., intravenous) and local (e.g., intratympanic or direct cochlear infusion) delivery has major implications for cell homing, survival, biodistribution, and therapeutic efficacy. Local delivery may offer higher targeted concentrations but is more invasive, while systemic administration is less invasive but may result in low engraftment in the auditory system. Perhaps the most profound challenge is assessing the long-term impact of such interventions on the already-formed “tinnitus trace” or maladaptive network within the central auditory system and associated limbic regions. Tinnitus is not merely a peripheral phenomenon but a persistent central neural signature. While trophic support might promote peripheral repair and reduce the drive for central gain, it is unclear whether it can actively reverse entrenched pathological neural circuits or if it merely prevents further deterioration. The success of pharmacological agents in normalizing central gain metrics, such as the auditory brainstem response wave V/I amplitude ratio, and reducing tinnitus behavior provides a benchmark for what cell therapies might need to achieve (Lu et al., 2021). Therefore, future research must not only optimize cell sourcing and delivery but also employ sophisticated behavioral and electrophysiological tools to determine whether paracrine-mediated repair can durably silence the phantom percept by fundamentally altering the stabilized tinnitus network. The molecular signaling pathways and druggable targets in tinnitus is shown in Figure 2.

## 9 Intervention strategies with direct clinical translation potential

While drug repurposing based on neurodegenerative disease mechanisms is a core focus of this research, several non-pharmacological or clinically applied interventions also show potential for tinnitus management by modulating these shared pathological pathways. These strategies are supported by preclinical evidence and, in many cases, already have a clinical evidence base or are being tested in ongoing trials.

### 9.1 Anti-inflammatory diet and nutritional supplements

Given that chronic neuroinflammation is a core pathological link between tinnitus and neurodegenerative diseases (Perin and Pizzala, 2024; He S. et al., 2025), dietary interventions aimed at modulating systemic and central inflammatory states represent a rational adjunctive strategy. For instance, natural compounds with anti-inflammatory and immunomodulatory properties have been investigated for neuroinflammatory conditions. Although direct clinical studies on anti-inflammatory diets for tinnitus are limited, the theoretical rationale is supported by evidence linking specific inflammatory proteins to tinnitus risk (He S. et al., 2025). Dietary interventions rich in bioactive components (e.g., polyphenols, antioxidants) may help mitigate the inflammatory milieu within the auditory and limbic systems by inhibiting microglial overactivation and downregulating pro-inflammatory signaling (Perin and Pizzala, 2024). Incorporating an anti-inflammatory dietary pattern (e.g., Mediterranean diet) as part of a comprehensive management plan is a safe lifestyle-based approach to target underlying inflammatory mechanisms.

### 9.2 Cognitive behavioral therapy (CBT)

Cognitive Behavioral Therapy is a first-line non-pharmacological treatment in current tinnitus clinical management (Perumareddi, 2025). Its mechanism of action aligns closely with modulating the maladaptive neuroplasticity and limbic system (emotional) dysfunction associated with tinnitus. CBT does not aim to eliminate the tinnitus sound but rather to reduce tinnitus-related distress and functional impairment by altering catastrophic cognitions and maladaptive behavioral responses to tinnitus (van der Wal et al., 2025). This constitutes a “behavioral-level” retraining and modulation of abnormal neural networks involving non-auditory brain regions such as the prefrontal cortex and amygdala. Its clinical efficacy is recognized, and it often serves as a control standard when evaluating novel therapies (Mazurek et al., 2025). CBT embodies a psycho-neurological intervention targeting the centralization and emotional components of tinnitus and is an indispensable element of integrated treatment (Jiang et al., 2025).

### 9.3 Neuromodulation techniques

Neuromodulation techniques, such as repetitive TMS, transcranial direct current stimulation (tDCS), and customized acoustic stimulation (e.g., “coordinated reset” therapy), aim to directly modulate the imbalanced cortical excitability and abnormal neural network oscillations implicated in tinnitus generation. These techniques apply external physical energy (magnetic, electrical, acoustic) to alter neuronal activity in specific brain regions (e.g., AC, dorsolateral prefrontal cortex), attempting to reverse chronic, pathologically enhanced neural synchrony. Research indicates that effective neuromodulation can induce objective changes in brain oscillatory activity (Fabrizio-Stover et al., 2025; Qian et al., 2025). A meta-analysis of randomized controlled trials suggests that rTMS has some efficacy in chronic tinnitus, although long-term effects require further validation (He Z. et al., 2025). The value of these methods lies in their direct and quantifiable mechanism of action via neurophysiological indices like electroencephalography (EEG) and their ongoing translation from research tools to clinical treatments, representing a crucial bridge connecting mechanistic research and clinical intervention (Mazurek et al., 2025).

## 10 Challenges in clinical translation and prospects for personalized therapy

### 10.1 Biomarker-driven patient stratification

Currently, the diagnosis of tinnitus relies almost entirely on subjective self-report, as no objective biomarker has been validated for clinical diagnosis or patient stratification. Therefore, any proposal to classify tinnitus patients into mechanistic subtypes based on biomarkers remains strictly hypothetical and is intended as a conceptual framework to guide future research, not as a current clinical tool. Nevertheless, inspired by the biomarker-based subtyping efforts in neurodegenerative diseases (Jack et al., 2024; Jack et al., 2016), we envision that future precision medicine for tinnitus could benefit from such stratification. This approach would acknowledge the heterogeneity of tinnitus pathophysiology, which, like many neurodegenerative conditions, likely involves multiple, overlapping mechanisms. Biomarker-driven stratification aims to move beyond a one-size-fits-all therapeutic model, enabling personalized interventions targeted at specific underlying pathological processes. The identification of objective, measurable biomarkers is therefore a critical prerequisite for such precision medicine in tinnitus. Research has identified a range of potential biomarkers across different domains. Neuroimaging studies offer promising cortical and subcortical morphological biomarkers. Machine learning applied to gray matter volume data from 61 brain regions identified a combination of 13 regions—including the bilateral hypothalamus, right insula, bilateral superior temporal gyrus, and left rostral middle frontal gyrus—that could differentiate tinnitus patients from healthy controls with an accuracy of 80% in a test dataset (Liu et al., 2019). This suggests distinct neuroanatomical signatures that could underpin different tinnitus subtypes. Functional neuroimaging also provides candidates; for instance, attention-modulated cortical responses, specifically the difference in N1 and P2 amplitudes between attended and unattended tones, were significantly greater in tinnitus subjects and showed potential as a diagnostic biomarker with 83.3% sensitivity and 76.9% specificity (Richardson et al., 2024). However, other resting-state quantitative EEG (qEEG) features, including spectral power and functional connectivity, showed no robust ability to differentiate tinnitus laterality after rigorous statistical correction, highlighting the challenge of identifying reliable electrophysiological biomarkers (Kim S. J. et al., 2025).

Beyond central nervous system markers, peripheral biomarkers are actively investigated. Circulating inflammatory proteins have been studied, though large-scale screening of 96 proteins did not reveal significant plasma biomarkers for constant tinnitus after multiple testing correction, and predictive models based on these inflammatory markers performed poorly (Cederroth et al., 2023). Conversely, a Mendelian randomization study suggested a causal relationship, identifying Chemokine (C-C motif) Ligand 19 (CCL19) as positively associated with tinnitus risk (Chen and Cai, 2025). Serum metabolomics has uncovered novel associations, with specific metabolites like homocitrulline and certain triglycerides and phosphatidylethanolamines being positively associated with persistent tinnitus, while others like *α*-keto-*β*-methylvalerate were inversely associated (Zeleznik et al., 2023). Furthermore, serum levels of the outer hair cell protein prestin, particularly the 97 kDa isoform, were elevated in tinnitus patients even after accounting for hearing loss and noise exposure, pointing to cochlear dysfunction as a potential biomarker for a “peripheral” subtype (Adamczyk et al., 2026). Other peripheral biomarkers include circulating proteasome activity in plasma, which was significantly lower in patients with chronic tinnitus and mild cognitive impairment, suggesting a link to cognitive comorbidity (Yun et al., 2020), and hair cortisol and BDNF levels, where hair cortisol is positively correlated with perceived tinnitus loudness, and hair BDNF is negatively correlated with both perceived tinnitus loudness and tinnitus-related distress (Basso et al., 2022). Auditory brainstem response (ABR) metrics, such as wave I amplitude and the V/I wave amplitude ratio, have also been proposed as audiological biomarkers for tinnitus presence and severity (Haider et al., 2022).

The stratification into mechanistic subtypes, such as “neuroinflammation-dominant,” could be guided by biomarkers like specific cytokines (e.g., associations found between interleukin-6 and tinnitus intensity (Tanaka et al., 2024)) or by functional imaging with specific tracers. For example, the utility of neuro-ophthalmological biomarkers like optical coherence tomography (OCT) in monitoring treatment response in idiopathic intracranial hypertension, a condition associated with pulsatile tinnitus, suggests the principle of using objective measures to track pathophysiological states (Hendrix et al., 2022). Similarly, identifying paraneoplastic etiologies, as in Kelch-like protein-11 (KLHL11) encephalitis where tinnitus can be a presenting symptom, underscores the importance of specific antibody biomarkers for patient stratification (Dubey et al., 2020). Ultimately, integrating multimodal biomarkers—combining neuroimaging, electrophysiology (Cardon et al., 2020), blood-based molecular profiles, and audiological measures—into a composite model holds the greatest promise. Such an approach, as demonstrated by a logistic regression model combining cortical auditory evoked potentials, brain signal variability, and cognitive scores, can significantly improve the objective detection of tinnitus (Cardon et al., 2022). This biomarker-driven framework is essential for future clinical trials and therapeutic development, allowing for the identification of patient subpopulations most likely to respond to targeted interventions, such as anti-inflammatory therapies, neuroplasticity modulators, or specific sound therapies.

**Figure 3:**
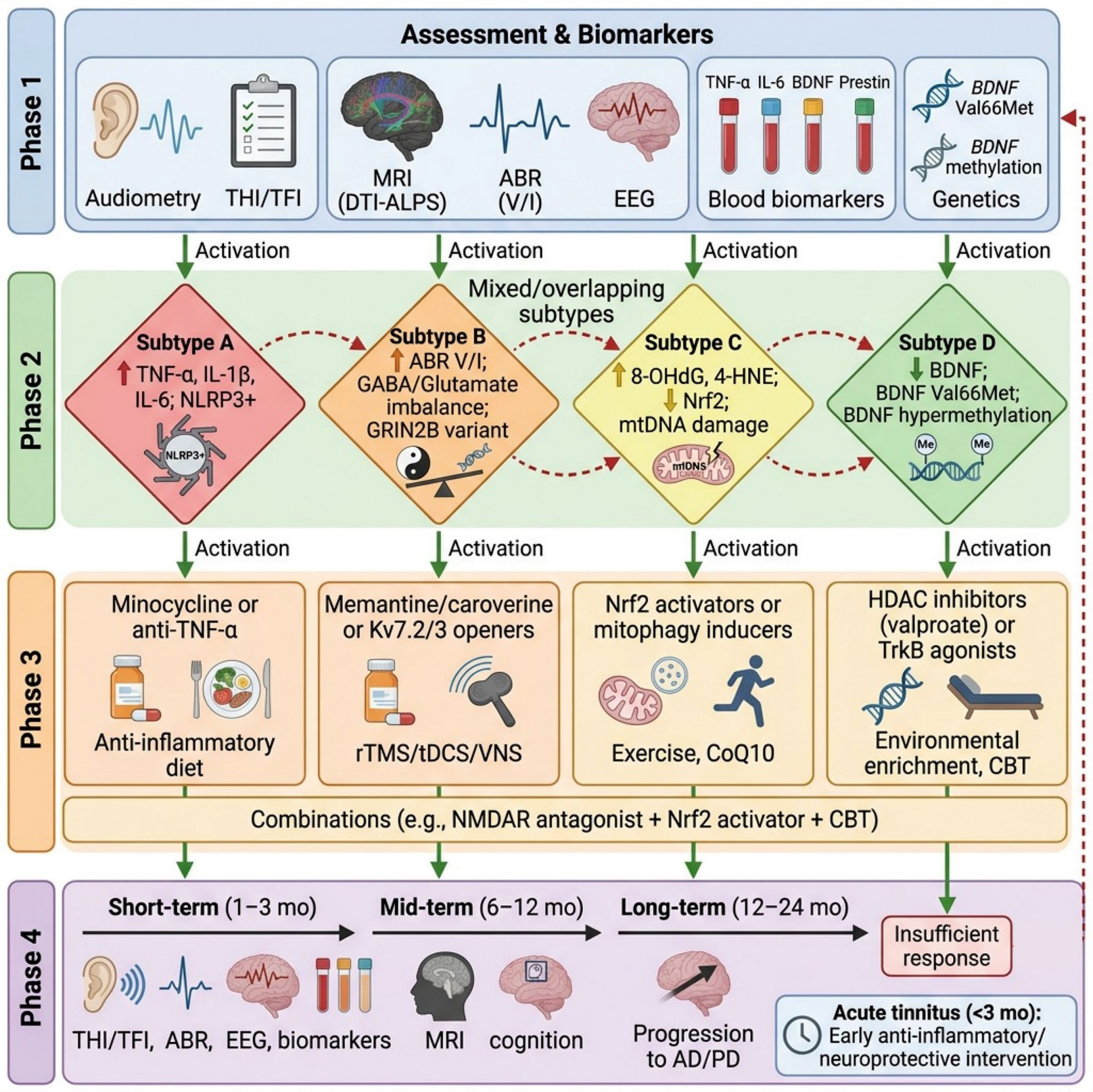
A proposed hypothetical biomarker-driven personalized treatment algorithm for tinnitus, a conceptual framework for future investigation. The four-phase framework integrates multimodal biomarker assessment (Phase 1), mechanistic subtype classification (Phase 2), targeted intervention (Phase 3), and long-term outcome monitoring (Phase 4). The classification into inflammation-dominant **(A)**, excitotoxicity-dominant **(B)**, oxidative-stress-dominant **(C)**, and epigenetic-neurotrophic **(D)** subtypes is illustrative and based on preclinical and correlational clinical evidence. The long-term follow-up in Phase 4 incorporates cognitive screening and neuroimaging to detect early neurodegenerative changes, given the reported epidemiological association between tinnitus and increased risk of Alzheimer’s and Parkinson’s disease. Dashed arrows indicate that mixed subtypes may require combination therapies. This schema is strictly speculative and designed to stimulate biomarker-enriched clinical trials; it does not represent a validated clinical protocol. This schema is speculative and intended for research purposes only; none of the depicted biomarkers or interventions have been validated for routine clinical stratification or treatment of tinnitus.

To synthesize these emerging concepts into a coherent research strategy, we propose a hypothetical, multi-phase framework for biomarker-driven personalized treatment of tinnitus, illustrated in Figure 3. Phase 1 builds on the multimodal assessment tools discussed above including audiometry, standardized questionnaires, neuroimaging, electrophysiology, and circulating molecular markers to capture the multi-dimensional profile of an individual patient. Phase 2 subclassifies patients into notional mechanistic subtypes (neuroinflammation-dominant, excitotoxicity-dominant, oxidative-stress-dominant, or neurotrophic-epigenetic-dominant) based on the predominant biomarker signatures, a concept directly derived from the stratification logic described in this section. Phase 3 assigns targeted interventions tailored to the putative subtype; these interventions correspond to the therapeutic strategies reviewed in Sections 4–9. Phase 4 outlines a longitudinal monitoring plan. Short-term follow-up (1–3 months) assesses treatment response with repeated questionnaires and electrophysiological measures; mid-term (6–12 months) tracks structural and cognitive changes; and long-term monitoring (12–24 months and beyond) aims to detect early signs of comorbid neurodegenerative conditions. The long-term component is motivated by cohort studies indicating that chronic tinnitus patients carry an increased risk of subsequently developing AD and PD (adjusted hazard ratios of 1.54 and 1.56, respectively) (Chu et al., 2020). Therefore, the goal of this phase is not to assert that tinnitus inevitably progresses to neurodegeneration, but rather to deploy cognitive screening and neuroimaging as part of a proactive surveillance strategy in at-risk populations. If treatment response is insufficient, the algorithm loops back to re-evaluate biomarkers and refine the subtype assignment. It must be emphasized that this framework is speculative and derived from preclinical and correlational clinical evidence. No validated biomarker panel currently exists to assign patients to these putative subtypes. The primary purpose of this schema is to stimulate hypothesis-driven research and the design of biomarker-enriched clinical trials, rather than to guide current clinical management.

### 10.2 Drug delivery and combination therapy strategies

The effective delivery of therapeutic agents to the auditory central nervous system and cochlea is a paramount challenge in tinnitus treatment, primarily due to the restrictive nature of the BBB and the analogous blood-labyrinth barrier (BLB) in the inner ear. These barriers severely limit the passage of most large-molecule drugs and systemically administered agents, necessitating innovative delivery technologies for successful clinical translation. Intratympanic (IT) administration has emerged as a primary local strategy to bypass systemic barriers, with recent advancements focusing on sustained-release formulations to overcome rapid drug clearance. For instance, a co-delivery system combining dexamethasone microcrystals and lidocaine-loaded poly (lactic-co-glycolic acid) (PLGA) non-spherical microparticles demonstrated prolonged drug retention in the perilymph and improved pharmacokinetics in guinea pig models, offering a promising approach for conditions like sudden SNHL associated with tinnitus (Wang et al., 2025). Similarly, the development of thermosensitive gels, such as AC102, for intratympanic injection has shown safety and tolerability in human trials, demonstrating the feasibility of this approach for inner ear drug delivery (Lanting et al., 2025). Beyond the middle ear, non-invasive trans-tympanic membrane delivery is being revolutionized by nanocarriers. Phosphatidylcholine-based liquid crystalline nanoparticles (PC-LCNPs) significantly enhanced the oto-topical delivery of caroverine across rabbit tympanic membranes compared to elastic vesicles, highlighting the potential of nano-sized carriers for non-invasive treatment (Farrah et al., 2020). Furthermore, liposomal nanoparticles, particularly transfersomes, have demonstrated the ability to penetrate the intact tympanic membrane and reach the inner ear compartments without causing hearing loss or hair cell damage, presenting a safe topical delivery mechanism (Kashfi Sadabad et al., 2022). For targeting the central auditory pathways, strategies must contend with the BBB. Nanomaterials, which have the characteristics of small size, high bioavailability, and strong plasticity, are a research hotspot in the field of inner ear diseases (Li et al., 2022). Innovative routes are also being explored, such as intranasal delivery of dexamethasone-loaded liposomal nanoparticles incorporated into a thermosensitive hydrogel (PLN-Dex@Gel). This approach utilizes dispersive transport via the brain and potentially the cochlear axis to reach the inner ear, showing efficacy in animal models of hearing loss (Ding et al., 2026). Physical methods to temporally modulate barrier permeability are also key. Focused ultrasound, especially when combined with microbubbles, is a promising non-invasive technique for acoustically mediated drug delivery through the round window membrane (Jeanneau et al., 2026). Research indicates that noise exposure itself can dynamically increase BLB permeability, creating a therapeutic window of 1–8 days post-exposure where systemic drug delivery to the cochlea is more efficient (Ke et al., 2024). Sound conditioning (SC) at specific parameters has also been shown to promote paracellular permeability of the BLB for up to 6 h, facilitating the entry of systemically administered drugs (Wang X. et al., 2024). These advanced delivery systems—encompassing nano-carriers, sustained-release depots, and barrier modulation technologies—are critical for translating neuroprotective and neuromodulatory agents from bench to bedside, ensuring that potentially therapeutic molecules reach their intended targets within the auditory system at effective concentrations.

Given the multifactorial and heterogeneous pathophysiology of tinnitus, which involves maladaptive neuroplasticity, inflammation, oxidative stress, and often comorbid psychological distress, a single-target pharmacological intervention is unlikely to be universally effective. This complexity mirrors that of neurodegenerative diseases like AD, where “cocktail” or multi-target combination therapies are increasingly seen as a necessary trend. A similar paradigm is emerging for tinnitus, advocating for the rational combination of agents with complementary mechanisms, potentially integrated with non-pharmacological interventions. Preclinical evidence supports this approach. For example, a combinatorial pretreatment with an antioxidant, a p53 inhibitor, and a neurotrophin provided significant multifactorial protection against cisplatin-induced ototoxicity *in vitro* and in cochlear explants, addressing concurrent biological mechanisms of cell death (Febles et al., 2022). This aligns with the concept of targeting multiple pathways, such as oxidative injury and apoptosis, which are implicated in drug-induced hearing loss and tinnitus. Similarly, magnetic cationic liposomes designed for targeted corticosteroid delivery reduced drug-induced cytotoxicity in hearing loss models by preserving mitochondrial function and reducing cellular senescence (Iftode et al., 2025), while liposome-encapsulated rutin attenuated cisplatin-induced ototoxicity by suppressing p53-associated oxidative injury (Liu et al., 2026). These studies exemplify the synergy possible in multi-agent delivery systems. Dexamethasone-loaded biopolymer-lipid hybrid microcarriers have been developed for inner ear delivery and sustained release (Dindelegan et al., 2022). Furthermore, the therapeutic regimen should extend beyond pharmacology. Given the strong cognitive and behavioral components of chronic tinnitus, the most effective multi-target intervention would likely integrate such a drug combination with established neuromodulatory and psychological therapies. For instance, the efficacy of targeted pharmacotherapy to reduce central gain or aberrant cortical oscillations—mechanisms implicated in tinnitus generation (Hayes et al., 2021)—could be potentiated by concurrent CBT to address maladaptive attentional and emotional responses. This integrated model of care, combining precision drug delivery for biological targets with behavioral intervention for perceptual and psychological components, represents a holistic and patient-centered approach. It acknowledges tinnitus as a symptom of both the ear and the brain, requiring a concerted attack on peripheral pathology, central neural plasticity, and the resultant conscious distress. The future of tinnitus treatment therefore lies not in a single magic bullet, but in rationally designed, personalized combination strategies that leverage advances in drug delivery to enable effective multi-target pharmacotherapy, seamlessly combined with sound-based and psychological therapies to achieve optimal patient outcomes.

### 10.3 Considerations for clinical trial design

The design of clinical trials for tinnitus interventions necessitates a critical evaluation of outcome measures and study duration to accurately capture therapeutic efficacy, particularly when evaluating strategies derived from neurodegenerative disease research. Tinnitus questionnaires are validated tools for assessing treatment response in tinnitus trials, and different types of tests each have their own advantages and disadvantages. While these instruments are validated and appropriate for measuring the psychological and functional impact of tinnitus, they present high within-individual variability. Systematic reviews have analyzed methodological pitfalls in tinnitus randomized controlled trials. Consequently, there is an urgent need to develop and validate more objective and reliable assessment endpoints. Promising avenues include the use of neurophysiological tools such as EEG or magnetoencephalography (MEG) to quantify tinnitus-related neural oscillations and cortical activity. For instance, studies have utilized EEG to demonstrate that effective interventions like tDCS can modulate cortical electrical activity, with changes in beta and theta frequency bands correlating with improvements in tinnitus perception (Souza et al., 2020). Similarly, a virtual reality-based intervention showed that treatment responders exhibited specific increases in theta and high beta band activity in the orbitofrontal cortex, as measured by standardized low-resolution brain electromagnetic tomography (sLORETA) analysis of EEG data (Park et al., 2022). Using validated test tools that match the purpose of the trial is essential to ensure the accuracy of results in tinnitus clinical trials.

Furthermore, the chronic and often progressive nature of tinnitus demands that clinical trials incorporate longer observation periods to assess an intervention’s potential to modify the disease course, rather than merely providing transient symptom relief. Systematic reviews have summarized and evaluated methodological limitations of tinnitus randomized controlled trials published since 2010. For example, a trial of tailor-made notched music training (TMNMT) versus tinnitus retraining therapy (TRT) assessed outcomes at 1 and 3 months, demonstrating efficacy but leaving the long-term trajectory unknown (Tong et al., 2023). In contrast, studies with extended follow-up have provided more clinically meaningful data. A large RCT of bimodal neuromodulation (sound and tongue stimulation) demonstrated not only significant reductions in THI and TFI scores after a 12-week treatment but also showed that therapeutic improvements were maintained or even continued for a full 12 months post-treatment for specific stimulation settings (Conlon et al., 2020). Similarly, a trial comparing real-time fMRI neurofeedback to CBT found that the neurofeedback group showed a significantly greater reduction in tinnitus distress at both 6-month and 12-month follow-ups, underscoring the value of longer-term assessment (Gninenko et al., 2024). The UNITI project, a large 12-week multi-center RCT, explicitly aims to compare single and combined treatments over a harmonized period (Schoisswohl et al., 2021). Incorporating longer observation periods, potentially spanning 12 to 24 months, is crucial for determining whether an intervention merely provides palliative relief or genuinely alters the maladaptive neuroplastic processes believed to underlie chronic tinnitus. This is especially pertinent for interventions targeting neuroprotection or synaptic stabilization, where the therapeutic goal is to halt or reverse progression, analogous to strategies in neurodegenerative diseases. Therefore, future trial designs must prioritize both the implementation of objective neurophysiological biomarkers and the adoption of extended follow-up schedules to comprehensively evaluate the true therapeutic potential and disease-modifying capacity of novel interventions for tinnitus.

## 11 Conclusion

The convergence of tinnitus and neurodegenerative diseases on shared pathological mechanisms, including chronic neuroinflammation, synaptic dysfunction, and excitotoxicity, has prompted a conceptual shift in understanding tinnitus not merely as a symptom but as a disorder of neural plasticity and resilience. This review has synthesized evidence that this mechanistic overlap provides a robust rationale for repurposing therapeutic strategies initially developed for neurodegenerative conditions. From an expert perspective, this represents a paradigm of translational medicine, where insights from one neurological domain can illuminate potential pathways in another, potentially accelerating therapeutic discovery. The development of this field underscores a move from viewing tinnitus through a purely auditory lens to recognizing it as a whole-brain network disorder with core neurodegenerative features, such as microglial priming, dysregulated neurotrophic support, and glutamate-mediated excitotoxicity.

Preclinical studies have compellingly demonstrated the efficacy of various strategies, including microglial modulation, neurotrophic factor regulation, and NMDA receptor antagonism, in alleviating tinnitus-like behaviors in animal models. These findings highlight significant translational potential. However, the journey from promising animal data to effective human therapies is fraught with substantial challenges that must be balanced against the initial optimism. The major hurdles—lack of objective diagnostic biomarkers, the BBB impeding drug delivery, significant patient heterogeneity, and the need for more sensitive clinical trial endpoints—collectively form a critical barrier to progress. An expert analysis must weigh the encouraging proof-of-concept from preclinical models against the sobering realities of clinical translation. For instance, while microglial inhibitors show promise in rodents, their systemic application in humans risks immunosuppression and lacks the precision needed for the specific neural circuits involved in tinnitus. Similarly, the patient heterogeneity observed clinically suggests that tinnitus is not a single entity but a final common pathway for various underlying dysfunctions, much like neurodegenerative syndromes. This necessitates a shift from a one-size-fits-all approach to a precision medicine framework.

Balancing these different research perspectives—the mechanistic optimism from preclinical science and the pragmatic constraints of clinical application—is essential for future advancement. The field must avoid the pitfall of premature, broad clinical trials based solely on animal efficacy, which have historically led to disappointing results in neurology. Instead, the focus should be on de-risking translation by addressing the identified challenges directly. Future research directions must therefore be strategically focused. First, leveraging multi-omics technologies and advanced neuroimaging is crucial to identify biologically defined tinnitus endophenotypes or subtypes. Such subtyping remains aspirational at present, but is an essential step toward a precision medicine paradigm in tinnitus. Just as AD research has progressed by distinguishing between amyloid and tau pathology, tinnitus research needs biomarkers that can stratify patients based on underlying mechanisms (e.g., predominant neuroinflammation vs. synaptic loss vs. central gain). This stratification is the cornerstone for personalized medicine.

Second, overcoming the BBB is a non-negotiable step for central nervous system-targeted therapies. Future efforts must prioritize the development of novel drug delivery systems, such as nanoparticle carriers, focused ultrasound, or intranasal delivery platforms, that can selectively target the auditory pathways and associated limbic regions (e.g., CN, IC, AC) with minimal off-target effects. This targeted delivery could enhance efficacy while reducing systemic side effects, making interventions more viable.

Finally, the goal should be the design of mechanism-based, personalized combination therapies. Given the multifactorial nature of tinnitus pathophysiology, monotherapies are unlikely to be universally effective. A rational approach might combine, for example, a peripherally acting agent to reduce cochlear damage with a centrally delivered anti-inflammatory agent and a cognitive-behavioral intervention to address the associated distress. This combinatorial strategy mirrors the approach in neurodegenerative diseases like Parkinson’s, where symptomatic relief is combined with neuroprotective strategies.

In conclusion, the exploration of neurodegenerative disease strategies for tinnitus represents a promising direction in the field. It bridges fundamental neuroscience with clinical otology, offering a more comprehensive pathophysiological framework. The impact of this line of inquiry lies in its potential to transition tinnitus management from purely symptomatic sound therapy or counseling towards true disease-modifying treatments that address the core neural pathology. Success will depend on a balanced, iterative process: using clinical observations to inform targeted preclinical models, and using mechanistic insights from those models to design smarter, biomarker-enriched clinical trials. By focusing on subtype identification, targeted delivery, and combination regimens, the field can navigate the current translational challenges and move closer to altering the disease course for millions of individuals affected by chronic tinnitus.
